# Molecular imaging of extracellular vesicles *in vitro via* Raman metabolic labelling†

**DOI:** 10.1039/d0tb00620c

**Published:** 2020-05-27

**Authors:** Conor C. Horgan, Anika Nagelkerke, Thomas E. Whittaker, Valeria Nele, Lucia Massi, Ulrike Kauscher, Jelle Penders, Mads S. Bergholt, Steve R. Hood, Molly M. Stevens

**Affiliations:** a Department of Materials, Imperial College London, London SW7 2AZ, UK; b Department of Bioengineering, Imperial College London, London SW7 2AZ, UK; c Institute of Biomedical Engineering, Imperial College London, London SW7 2AZ, UK; d GSK Medicines Research Centre, Gunnels Wood Road, Stevenage, Hertfordshire SG1 2NY, UK

## Abstract

Extracellular vesicles (EVs) are biologically-derived nanovectors important for intercellular communication and trafficking. As such, EVs show great promise as disease biomarkers and therapeutic drug delivery vehicles. However, despite the rapidly growing interest in EVs, understanding of the biological mechanisms that govern their biogenesis, secretion, and uptake remains poor. Advances in this field have been hampered by both the complex biological origins of EVs, which make them difficult to isolate and identify, and a lack of suitable imaging techniques to properly study their diverse biological roles. Here, we present a new strategy for simultaneous quantitative *in vitro* imaging and molecular characterisation of EVs in 2D and 3D based on Raman spectroscopy and metabolic labelling. Deuterium, in the form of deuterium oxide (D_2_O), deuterated choline chloride (d-Chol), or deuterated D-glucose (d-Gluc), is metabolically incorporated into EVs through the growth of parent cells on medium containing one of these compounds. Isolated EVs are thus labelled with deuterium, which acts as a bio-orthogonal Raman-active tag for direct Raman identification of EVs when introduced to unlabelled cell cultures. Metabolic deuterium incorporation demonstrates no apparent adverse effects on EV secretion, marker expression, morphology, or global composition, indicating its capacity for minimally obstructive EV labelling. As such, our metabolic labelling strategy could provide integral insights into EV biocomposition and trafficking. This approach has the potential to enable a deeper understanding of many of the biological mechanisms underpinning EVs, with profound implications for the design of EVs as therapeutic delivery vectors and applications as disease biomarkers.

## Introduction

Most, if not all, cells secrete extracellular vesicles (EVs).^1^ These are nanoscale entities comprising a lipid bilayer that encases an aqueous core and contains a wealth of biological materials, such as proteins, RNA, and miRNA. EVs consist of a population of vesicles that is highly heterogeneous not only in size, but also in composition, and this is reflected in their diverse origins and the variety of biological roles they are thought to play in a host of cellular processes such as cell–cell signalling and nucleic acid transport.^2,3^ Due to their purported biological significance, much effort has been paid to the development of EVs as both diagnostic biomarkers and therapeutic delivery vectors.^4–9^ However, while many promising results have been achieved, there remains much uncertainty about their innate biological roles, transportation and uptake mechanisms, and clinical potential.^10,11^

In this respect, characterising how EVs interact with target cells is vital to fully optimise their use as next generation therapeutics.^12^ Previous EV imaging work has relied heavily on fluorescence microscopy to study the intracellular uptake and biodistribution of EVs. To date, this has been achieved through labelling with lipophilic dyes,^13,14^ surface functionalisation *via* click chemistry,^15,16^ or genetic engineering of cells to express fluorescent proteins fused to EV-associated markers.^17,18^ However, while these strategies have enabled the *in vitro* visualisation of EVs, there remain concerns about the specificity and stability of fluorescent dye labelling and the impacts of EV surface modifications on their biological functions.^19–23^ Further, fluorescent labelling strategies provide no information on the molecular composition of EVs or insight into compositional changes that might occur in the context of different biological processes. As such, there is a clear need for *in vitro* imaging and molecular characterisation strategies that do not interfere with the native functionality of EVs. These will help gain a deeper understanding of the mechanisms underpinning their functionality towards the development of EV therapeutic systems.

Raman spectroscopy is one such molecular imaging technique that has received increasing attention as a highly sensitive tool for studying complex biological systems including cells, tissues, and various biomaterials.^24–28^ Raman spectroscopy interrogates the biochemical constituents of a sample through the collection of inelastically scattered light by different molecular species. In this way, it provides a fingerprint spectrum for a given sample on the basis of the chemical bonds present within.^25^ Crucially, Raman spectroscopy can be applied to aqueous samples, enabling non-destructive, label-free spectroscopic imaging and analysis of cells, tissues, and nanoparticles.^29–31^ While much information can be gleamed from label-free spectroscopic imaging of cells, detailed analysis of particular sub-cellular structures (*e.g.* EVs) can be confounded by the similarity of spectral signatures within cells due to the large number of chemical bonds in common across these structures.^31–33^

To circumvent this, numerous studies have investigated the use of small bio-orthogonal Raman tags, akin to fluorescent labelling, that vibrate in the silent region (1800–2800 cm^−1^) of the Raman spectrum.^34–39^ In this region, no endogenous biological molecules display Raman signatures, enabling unobstructed spectroscopic imaging of molecules or structures labelled with such Raman tags.^40,41^ This approach has previously been successfully applied to the visualisation and tracking of the cellular uptake of various liposomal drug delivery systems using Raman spectroscopy.^38,42^ Importantly, these Raman tags, the most common of which are alkyne (C≡C) and carbon-deuterium (C–D) bonds, are much smaller than fluorescent tags and therefore anticipated to have a much less significant effect on the structure and function of the molecules under investigation.^32,33,41,43^ Deuterium in particular, despite having a Raman cross-section that is an order of magnitude lower than alkynes, can be stably incorporated into multiple locations (replacing C–H bonds) within a single molecule without impacting physiological function.^33,36^ Raman spectroscopy has recently been applied to the characterisation of EVs isolated from cells, with both bulk measurements and single-EV measurements used for detailed compositional characterisation.^44–47^ The capacity of Raman spectroscopy to both identify the cellular origin of EVs and discern subpopulations that exist across cell types has been demonstrated, with potential applications in the characterisation of EVs prior to therapeutic use.^44^ However, while the application of Raman spectroscopy in this way could prove useful for the characterisation of EVs prior to therapeutic application, it does not allow for *in vitro* characterisation of EVs upon cellular uptake.

Here, we demonstrate a minimally obstructive metabolic EV labelling strategy for Raman spectroscopic imaging in 2D and 3D. Using three deuterium-labelled metabolites, namely deuterium oxide (D_2_O), deuterated choline chloride (d-Chol), and deuterated D-glucose (d-Gluc), as Raman-active biorthogonal tags, we illustrate the metabolic incorporation of deuterium into EVs to enable simultaneous quantitative Raman spectroscopic visualisation of EV distribution *in* *vitro* as well as comprehensive molecular characterisation of EVs both in solution and *in vitro*. We show that metabolic labelling of EVs with deuterium is stable, nontoxic, and has limited effect on their chemical composition. Simultaneous *in vitro* visualisation and compositional analysis of metabolically labelled EVs *via* Raman spectroscopy thus offers exciting new opportunities to gain a deeper understanding of their composition, trafficking, and fate upon cellular uptake with significant implications for the design and development of EVs as therapeutic delivery vectors.

## Materials and methods

### Cell culture

MDA-MB-231 breast cancer cells and MCF10A non-malignant breast epithelial cells were originally obtained from the ATCC (Manassas, VA, USA) and authenticated *via* STR profiling. MDA-MB-231 cells were maintained in Dulbecco’s modified Eagle’s medium (DMEM), supplemented with 1× non-essential amino acids, 25 mM Hepes, 1× penicillin/streptomycin, and, where indicated 10% (v/v) foetal bovine serum (FBS), all obtained through Gibco (Thermo Fisher Scientific, Inc., Waltham, MA, USA). MCF10A cells were maintained in DMEM/F12 (Gibco), supplemented with 5% horse serum (Gibco), 20 ng mL^−1^ epidermal growth factor (EGF, PeproTech, London, UK), 0.5 μg mL^−1^ hydrocortisone (Sigma Aldrich, St. Louis, MO, USA), 100 ng mL^−1^ cholera toxin (Sigma), 10 μg mL^−1^ insulin (Sigma) and 1× penicillin/streptomycin (Gibco). Both cell lines were cultured at 37 °C and 5% CO_2_.

### Metabolic labelling

For metabolic labelling, the standard cell culture medium was adapted as follows: (i) supplementation with 1 mM deuterated choline chloride (492051, Sigma), (ii) substitution of _D_-glucose with 50% (w/w, or 2.25 g L^−1^) deuterated _D_-glucose (552003, Sigma), and (iii) substitution of H_2_O with 50% (v/v) deuterium oxide (D_2_O, VWR International Ltd, Lutterworth, UK).

### Cell survival assay

To assess the effects of metabolic labelling on cell survival, MDA-MB-231 cells were seeded in 96-wells plates at 31 250 cells per cm^2^ in serum-supplemented DMEM and allowed to adhere overnight. Cell culture medium was carefully aspirated cells were washed once in Dulbecco’s phosphate-buffered saline (DPBS, Gibco), after which serum-free DMEM with or without metabolic labelling components was added. After 72 hours of incubation, cell culture medium was carefully aspirated and cells were washed once in DPBS, after which serum-supplemented DMEM was added to all conditions for another 72 hours. Cell survival was determined using the cell counting kit 8 (CCK8) assay from Sigma, according to the instructions provided.

### Cell culture for EV production

To maximize EV yield, EVs were collected from MDA-MB-231 cultures, grown non-adherent in 125 mL Erlenmeyer flasks on an orbital shaker at 125 rpm. Cells were cultured at a starting concentration of 1 × 10^6^ cells per mL in serum-free medium for 72 hours, before conditioned medium was harvested and cells discarded. Conditioned medium was filtered using 0.45 μm bottle top filters (VWR, UK) and stored at −80 °C until further processing.

### EV purification

Conditioned medium was concentrated using ultrafiltration with Amicon Ultra-15 centrifugal filter units (EMD Millipore, Burlington, MA, USA). A maximum of 100 mL of conditioned medium was used per filter unit. Concentration was performed by centrifugation at 5000 × *g* until 0.5 mL of volume remained. EVs were isolated from concentrated conditioned medium using size exclusion chromatography (column length 30 cm and diameter 1 cm) with Sepharose CL-2B resin (Sigma). DPBS was used as the mobile phase and 24 consecutive fractions of 1 mL were collected. EV positive fractions (as determined with Nanoparticle Tracking Analysis and immunoblotting) were pooled and stored at −80 °C until successive experiments.

### Nanoparticle tracking analysis (NTA)

EV concentration was measured on a Nanosight NS300 (Malvern Panalytical Ltd, Malvern, UK) equipped with a 532 nm laser and sCMOS camera. EVs were diluted in sterile DPBS to a concentration of 10^8^–10^9^ particles per mL. Using NTA V3.0 software, at least three 30 second videos were recorded in different fields with a camera level of 15 and analysed with a detection threshold of 5.

### Protein concentration measurements

Protein concentrations were determined using the BCA Protein Assay Kit (Thermo). 10 μL sample was mixed with 200 μL working reagent, consisting of 49 parts A and 1 part B, and subsequently incubated at 37 °C for 30 minutes. Absorbance was measured at 562 nm on a Spectramax M5 (Molecular Devices, San Jose, CA, USA).

### Immunoblotting: western blotting

Protein lysates were prepared from both cells and EVs. Cells and concentrated EVs were lysed using RIPA buffer (Cell Signaling Technology, Danvers, MA, USA), supplemented with phosphatase and protease inhibitors (Roche, Basel, Switzerland). Lysates were sonicated with a VibraCell VCX500 sonicator (Sonics & Materials Inc., Newtown, CT, USA) for 20 s at 20% amplitude on ice, followed by a 1 hour incubation at 4 °C with gentle mixing. Samples were centrifuged for 10 minutes at 20 000 × *g* and the pellets discarded. Protein was mixed with Laemmli sample buffer (Bio-Rad Laboratories, Inc., Hercules, CA, USA) without reducing agent, separated by SDS-PAGE on Criterion XT Precast 4–12% Bis–Tris gels (Bio-Rad) and blotted onto PVDF membranes (EMD Millipore). Membranes were blocked in 5% (w/v) nonfat dry milk (Bio-Rad) in TBS with 0.1% (v/v) Tween-20 (Sigma) for 1 hour at room temperature, washed three times 10 minutes in TBS-T and incubated overnight at 4 °C in one of four primary antibodies: mouse-anti-CD9, (Thermo, 10626D), mouse-anti-CD63, (Thermo, 10628D), mouse-anti-CD81, (Thermo, 10630D) or rabbit-anti-calnexin (Cell Signaling Technology, #2679) all diluted 1 : 1000 in 5% (w/v) bovine serum albumin (BSA, Sigma) in TBS-T. The next day, membranes were washed three times 10 minutes in TBS-T and incubated for 1 hour at room temperature with LiCor-dye 800 CW-conjugated goat-anti-mouse or goat-anti-rabbit (LiCor Biosciences) secondary anti-body diluted 1 : 10 000 in TBS-T. After another three 10 minute washes in TBS-T, membranes were imaged on a LiCor Odyssey near-infrared imager (LiCor).

### Immunoblotting: dot blotting

100 μL of each column fraction (24 in total) was applied to a 0.45 μm nitrocellulose membrane (Bio-Rad), prewetted in Tris-buffered saline (TBS), in a BioDot apparatus (Bio-Rad). Membranes were blocked in 5% (w/v) nonfat dry milk (Bio-Rad) in TBS with 0.1% (v/v) Tween-20 (Sigma) for 1 hour at room temperature, washed three times 10 minutes in TBS-T and incubated overnight at 4 °C in one of three primary antibodies: mouse-anti-CD9, (Thermo, 10626D), mouse-anti-CD63, (Thermo, 10628D), or mouse-anti-CD81, (Thermo, 10630D) all diluted 1 : 1000 in 5% (w/v) bovine serum albumin (BSA, Sigma) in TBS-T. The next day, membranes were washed three times 10 minutes in TBS-T and incubated for 1 hour at room temperature with LiCor-dye 800 CW-conjugated goat-anti-mouse (LiCor Biosciences) secondary antibody diluted 1 : 10 000 in TBS-T. After another three 10 minute washes in TBS-T, membranes were imaged on a LiCor Odyssey near-infrared imager (LiCor). Blots were quantified using the LiCor ImageStudio software.

### Cryogenic transmission electron microscopy (Cryo-TEM)

Samples for Cryo-TEM were prepared using an automatic plunge freezer (Leica EM GP), in which 4 μL was spotted on plasma-treated (O2/H2 for 15 seconds) HoleyCarbon copper grids in an environmental chamber (relative humidity: 90%, temperature: 20 °C). Excess suspension was blotted onto filter paper and the obtained film was vitrified in liquid ethane. Samples were stored in liquid nitrogen and imaged at −170 °C (Gatan cryo-holder) in a JEOL 2100Plus transmission electron microscope. Micrographs were taken at 200 kV in low electron dose mode.

### Cell culture for Raman spectroscopy

For analysis with Raman spectroscopy, cells were grown on magnesium fluoride windows (Global Optics Ltd, Bournemouth, UK). 20 000 cells per cm^2^ were seeded in serum-supplemented DMEM and allowed to adhere overnight. Experiments were performed in serum-free conditions. For cell uptake experiments of EVs, purified EVs were mixed 1 : 1 with 2× DMEM (Gibco) and filtered sterile with 0.45 μm syringe filters (Corning Inc. New York, NY, USA). Upon completion of the experiments and prior to imaging, cells were washed with DPBS and fixed with 4% (v/v) paraformaldehyde (PFA, Sigma) in DPBS for 30 minutes at room temperature. PFA solution was aspirated and samples washed three times with DPBS.

### Raman spectroscopic cellular imaging

Raman imaging of MDA-MB-231 cells was performed using a confocal Raman microscope (alpha300R+, WITec, GmbH, Germany). A 532 nm laser light source at 35 mW power output was applied through a 63×/1.0 NA water-immersion microscope objective lens (W Plan-Apochromat, Zeiss, Germany). Inelastically-scattered light was collected through the objective and directed *via* a 100 μm diameter silica fibre, acting as a confocal pinhole, to a high-throughput imaging spectrograph (UHTS 300, WITec, GmbH, Germany) using a 600 groove per mm grating and equipped with a thermoelectrically cooled (−60 °C) back-illuminated charge-coupled device (CCD) camera. Raman spectra were acquired in the range from 0 to 3700 cm^−1^ with a spectral resolution of 11 cm^−1^. PFA-fixed cells on MgF_2_ windows were imaged with 500 nm resolution and 1 second integration time. Spectral image processing was performed using Witec ProjectFOUR software. Briefly, spectra were first cropped to remove laser contribution. Background autofluorescence subtraction was performed using a ‘shape’ background filter with parameter size 500 and spectra normalised to the area under the curve. System spectral background subtraction was then performed using spectra from the exterior of the cell, prior to analysis. Univariate image analysis was then performed by calculating the total area under the curve for different spectral features, namely: 985–1015 cm^−1^ (‘nucleoli’), 775–805 cm^−1^ (‘nuclei’), 1425–1485 cm^−1^ (‘lipids’), 1635–1685 cm^−1^ (‘proteins’), and 2800–3000 cm^−1^ (‘whole cell’). Univariate analysis of deuterium content was then calculated according to the full width at half maximum (FWHM) of the peak between 2025–2275 cm^−1^, where a sharper peak corresponds to a greater relative deuterium content.

### Raman spectroscopic extracellular vesicle trapping analysis

Pooled extracellular vesicle fractions isolated from cells were incubated at 37 °C, 5% CO_2_ for 0, 1, 2, 4, 8, or 24 hours and then placed at −80 °C prior to Raman analysis. Extracellular vesicle solutions were diluted to obtain a clear solution such that trapping efficiency was approximately 10%; corresponding to particle concentrations approximately between 1 × 10^10^–1 × 10^12^ particles per millilitre. 100 μL of extracellular vesicle solution was placed onto a 22 mm coverslip affixed to a standard microscopy slide with a drop of phosphate buffered saline (DPBS). The sample was then placed underneath the 63×/1.0 NA water-immersion objective and measured using the confocal Raman spectroscopy system described above. Individual EVs were trapped using a previously described method.^29^ Spectra of individually trapped EVs were acquired with a 5 second integration time, with the laser manually disabled for at least 5 seconds between acquisitions to enable diffusion of the previously trapped EVs. The deuterium content of each extracellular vesicle was determined by calculating the ratio of the peak intensity at 2140 cm^−1^ to the peak intensity at 1440 cm^−1^, corresponding to the ratio of carbon–deuterium (C–D) bonds to carbon–hydrogen (C–H) bonds in the EVs.

### Raman spectroscopic image processing and analysis

Automated Raman spectroscopic image processing and analysis was performed using a custom-built script in MATLAB. Previously generated univariate whole cell and deuterium images were overlaid to identify deuterium signal present inside and outside the cell. Deuterium cell content and the distances between each deuterium-positive pixel and the nearest segment of cell membrane were calculated to quantify EV uptake. Raman spectral signatures for deuterium-positive pixels were extracted and analysed *via* PCA using PLS_Toolbox (Eigenvector Research).

## Results and discussion

**Fig. 1:**
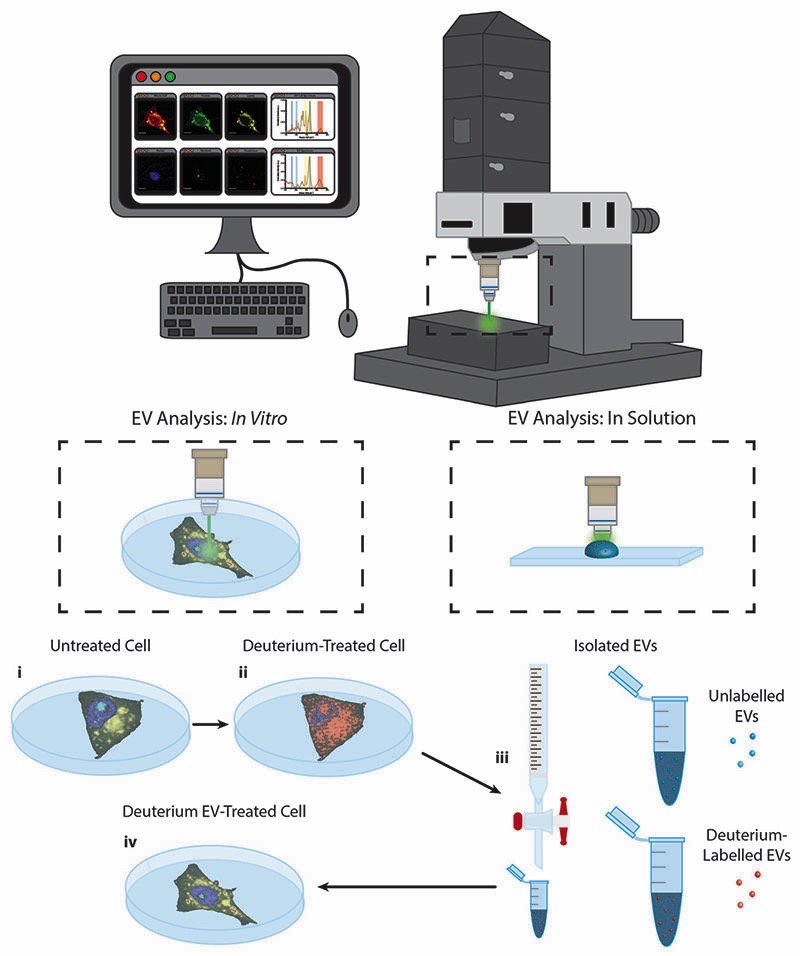
Raman spectroscopic imaging and analysis of metabolically labelled EVs in solution and *in vitro*. Illustration of metabolic labelling and Raman spectroscopic analysis of cells and EVs using the confocal Raman microspectroscopy system; (i) cells cultured on MgF_2_ windows are imaged *via* Raman microspectroscopy (control cells) or (ii) are incubated further with deuterium-containing (D_2_O, d-Chol, or d-Gluc) cell culture medium and imaged *via* Raman microspectroscopy (deuterium-treated cells) or (iii) the EVs are isolated from the cells and assessed *via* Raman trapping analysis or (iv) the metabolically deuterium-labelled EVs are incubated with unlabelled cells before cell imaging *via* Raman microspectroscopy (deuterium EV-treated cells).

In this work we present the Raman spectroscopic analysis of metabolically labelled EVs and their subsequent cellular uptake. An overview of the study is given in Fig. 1. Raman spectroscopy was performed using a confocal spontaneous Raman microspectroscopy system. This system enables both spectral compositional analysis of isolated and optically-trapped EVs in solution^29^ as well as high resolution spectroscopic imaging of cells and EV uptake *in vitro* in 2D and 3D.

To achieve Raman spectroscopic imaging of EVs, we first optimized our procedures for isolation of EVs from cell culture supernatants, using serum starved MDA-MB-231 breast cancer cells. We used ultrafiltration to concentrate the conditioned medium, followed by size exclusion chromatography (SEC) to separate EVs from other components. Fig. S1 (ESI†) shows the column trace of the material collected from the SEC for particle number as determined by Nanoparticle Tracking Analysis (NTA), protein quantities and EV marker expression (ESI,† Fig. S1a–c). Together, these results show the separation of particles, expressing EV markers from free protein in later column fractions. We next pooled the EV-containing fractions and assessed morphology using cryo-transmission electron microscopy (cryo-TEM, ESI,† Fig. S1d) and marker expression using Western blotting (ESI,† Fig. S1e). We observed vesicular structures in the cryo-TEM analysis, indicating the presence of EVs. Furthermore, analysis of EV markers showed enrichment for CD9, CD63 and CD81 expression in EVs compared to their parent cells, whereas the endoplasmic reticulum marker Calnexin was only observed in cells, not EVs. This is in accordance with the MISEV guidelines.^48^

**Fig. 2:**
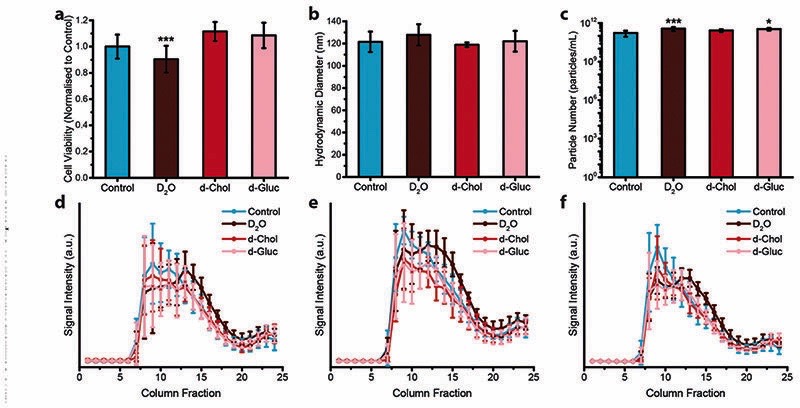
Effect of deuterium labelling on cell viability and extracellular vesicle production. (a–c) Effect of deuterium labelling on (a) cell viability (*N* = 2, *n* = 12), (b) hydrodynamic diameter of isolated EVs (*N* = 6, *n* = 3), and (c) particle number of isolated EVs (*N* = 6, *n* = 3) for MDA-MB-231 cells cultured on deuterium-containing medium (containing D_2_O, d-Chol, or d-Gluc) for 72 hours as compared to untreated controls, measured *via* nanoparticle tracking analysis. Data presented as mean SD ± (one-way ANOVA, Tukey’s honest significant differences *post hoc* correction, *P < 0.05, **P < 0.01, ***P < 0.001). (d–f) Expression of three different EV markers, (d) CD9, (e) CD63, and (f) CD81, for EVs isolated from MDA-MB-231 cells cultured on deuterium-containing medium (containing D_2_O, d-Chol, or d-Gluc) for 72 hours as compared to untreated controls measured *via* immunoblotting analysis (*N* = 6, *n* = 1). Data presented as mean ± SD.

Next, we assessed the effect that deuterium metabolic labelling has on cell viability as well as EV production and composition, given the known low toxicity that deuterium displays towards animal cells.^49^ MDA-MB-231 cells were cultured in the presence of deuterium oxide (D_2_O), deuterated choline chloride (d-Chol), or deuterated _D_-glucose (d-Gluc) for 72 hours. Cells cultured in cell culture medium containing d-Chol or d-Gluc did not display a statistically significant reduction in cell viability relative to cells cultured in cell culture medium without the addition of a deuterated compound, while cells cultured in cell culture medium containing D_2_O showed a small, but statistically significant, decrease in viability (Fig. 2a). We next assessed the effects of incubating the cells in deuterium-containing cell culture medium on EV production. MDA-MB-231 cells were cultured in medium with or without deuterated compounds. Subsequently, EVs were isolated from the conditioned medium. We performed NTA to assess the effect of metabolic labelling on EV size and quantity. The hydrodynamic diameter of the particles showed no difference between the different conditions (Fig. 2b). No statistically significant change was observed in the number of EVs with the exception of a small, but statistically significant increase, in the number of EVs from D_2_O treated cells relative to EVs isolated from control cells (Fig. 2c). Using cryo-TEM analysis, vesicle morphology was studied. No obvious morphological changes were observed in EVs isolated from cells cultured under each condition (ESI,† Fig. S2). We further investigated whether deuterium metabolic labelling had an effect on the expression of three EV protein markers CD9, CD63, and CD81. Protein marker expression was confirmed *via* immunoblotting analysis, with expression profiles for each column fraction following similar trends for each of the culturing conditions (Fig. 2d–f). Together, these results demonstrate that metabolic labelling with each of the deuterated compounds investigated does not affect the size, number, or marker expression of the EVs produced, indicating the suitability of this method as a minimally obstructive labelling strategy. However, analysis of the effects of deuterium labelling on the complex biological functions of EVs will require further studies.

**Fig. 3:**
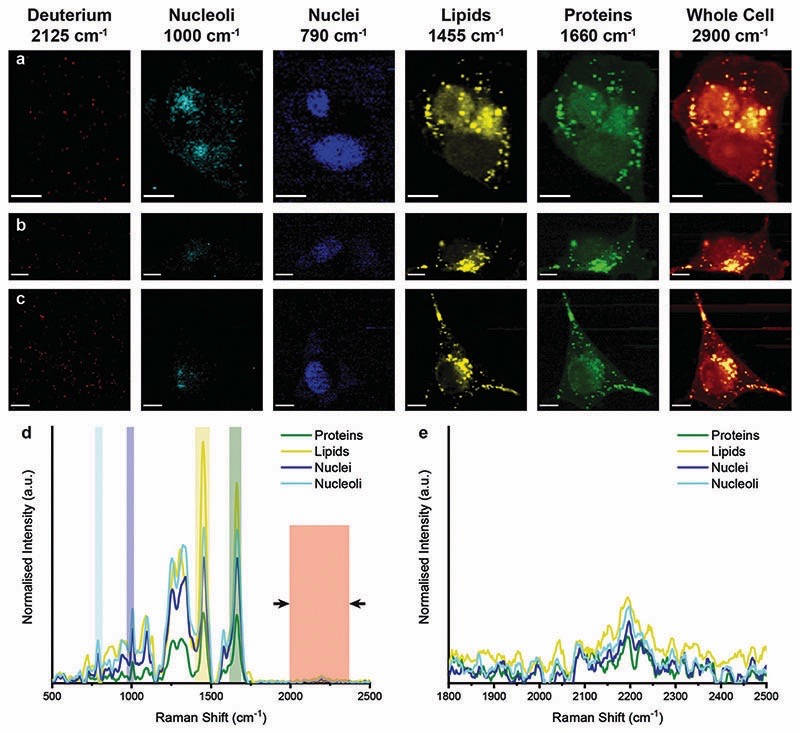
Raman spectroscopic imaging and univariate analysis of untreated MDA-MB-231 cells. (a–c) Univariate images of normalised Raman spectroscopic data for untreated MDA-MB-231 cells (*n* = 3) highlighting the distribution of different subcellular components including deuterium (red, 2025–2275 cm^−^^1^), nucleoli (cyan, 985–1015 cm^−^^1^), nuclei (blue, 775–805 cm^−^^1^), lipids (yellow, 1425–1485 cm^−^^1^), proteins (green, 1635–1685 cm^−^^1^), and the whole cell (2800–3000 cm^−^^1^). Scale bars = 10 μm. (d) Exemplar Raman spectra and (e) magnified silent region of cell areas positive for nucleoli, nuclei, lipids, and proteins (arrows indicate FWHM peak measurement, see Materials and methods for details).

We next analysed metabolic labelling of the cells using Raman spectroscopy. We first performed 0.5 μm spatial resolution Raman spectroscopic imaging of control MDA-MB-231 cells incubated in deuterium-free cell culture medium to demonstrate both label-free imaging of subcellular components and an absence of deuterium signal (Fig. 3). Univariate analysis of specific Raman peaks facilitated imaging of multiple sub-cellular components as well as the metabolically labelled EVs. Here, univariate analysis represents a simplified representation of the complex, information-rich Raman spectra – useful to make visual comparisons to existing techniques such as confocal fluorescence microscopy. Univariate identification of sub-cellular components was achieved using tentative spectral band assignments of 775–805 cm^−1^ for phosphodiester bond stretching in DNA (nuclei),^50,51^ 985–1015 cm^−1^ for phenylalanine (nucleoli),^52^ 1425–1485 cm^−1^ for CH_2_ bending (lipids),^53^ 1635–1685 cm^−1^ for the amide I region and CQC stretching (proteins),^54^ 2025–2275 cm^−1^ for C–D vibrations (deuterium),^37^ and 2800–3000 cm^−1^ for CH, CH_2_, and CH_3_ vibrations in proteins and lipids (whole cell).^51^ Due to the many chemical species in common across these biological components, their Raman spectra appear quite similar. This is particularly the case for lipid and protein signatures. However, the high specificity of Raman spectroscopy enables ready distinction between components *via* univariate analysis due to variations in peak ratios, *etc.*, while more complex multivariate analyses and machine learning techniques can be applied to gain deeper insights into the high information content Raman spectra. As expected, no deuterium signal was observed in the unlabelled control MDA-MB-231 cells (Fig. 3).

**Fig. 4:**
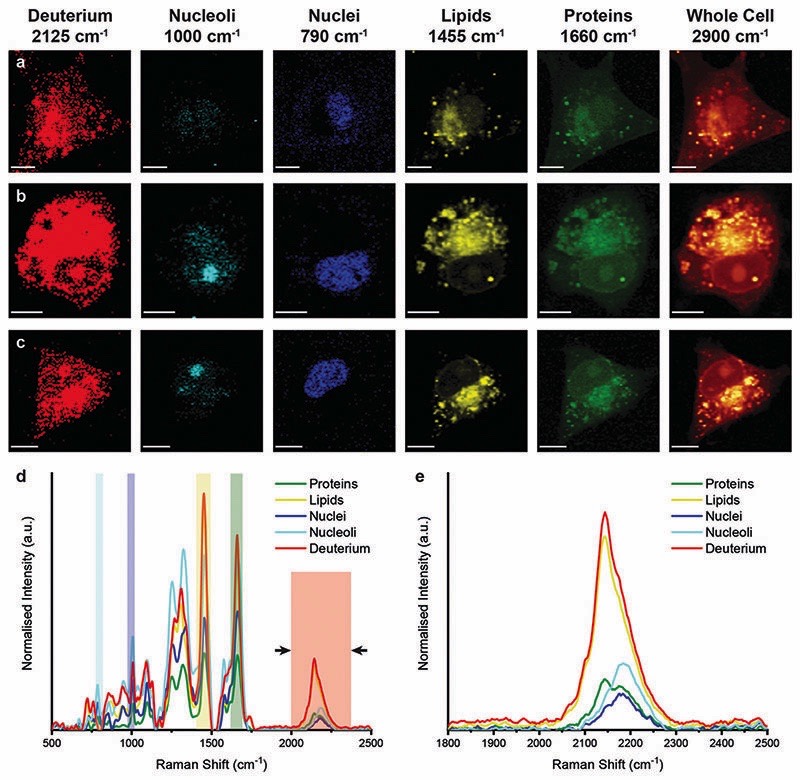
Raman spectroscopic imaging and univariate analysis of D_2_O-treated MDA-MB-231 cells. (a–c) Univariate images of normalised Raman spectroscopic data for MDA-MB-231 cells (*n* = 3) after incubation in D_2_O-containing medium for 72 hours highlighting the distribution of different subcellular components including deuterium (red, 2025–2275 cm^−^^1^), nucleoli (cyan, 985–1015 cm^−^^1^), nuclei (blue, 775–805 cm^−^^1^), lipids (yellow, 1425– 1485 cm^−^^1^), proteins (green, 1635–1685 cm^−^^1^), and the whole cell (2800–3000 cm^−^^1^). Scale bars = 10 mm. (d) Exemplar Raman spectra and (e) magnified silent region of cell areas positive for deuterium, nucleoli, nuclei, lipids, and proteins (arrows indicate FWHM peak measurement, see Materials and methods for details).

We then performed Raman spectroscopic imaging and univariate spectral analyses on MDA-MB-231 cells incubated in cell culture medium containing either D_2_O, d-Chol, or d-Gluc for 72 hours (Fig. 4 and ESI,† Fig. S3, S4). In each case, a clear deuterium signal between 2025 and 2275 cm^−1^ was observed throughout the cell, with subtle differences in the location of the main deuterium peak and with the appearance of additional peaks in the d-Chol sample owing to the different deuterium environments of the compound (ESI,† Fig. S3 and S4).^55^ These subtle differences provide insight into the metabolism of each of these compounds, with high deuterium content detected in protein-rich regions for d-Gluc cells as opposed to predominantly lipid-rich regions for d-Chol cells, presenting a potential avenue to exploit and identify different EV biogenesis pathways. The detected deuterium signal was most intense and prevalent for cells incubated in D_2_O-containing medium. Using a single particle automated Raman trapping analysis technique we recently developed for EV-sized particles,^29^ we also confirmed that the highest deuterium signal was observed in EVs isolated from cells incubated in D_2_O-containing medium (ESI,† Fig. S5). We therefore focussed on D_2_O-labelled EVs for *in vitro* imaging and molecular characterisation studies.

To determine whether we could observe and characterise metabolically labelled EVs *in vitro* using confocal Raman spectroscopic imaging, we first assessed the stability of the metabolic labelling of EVs with D_2_O under cell culture conditions. Isolated D_2_O EVs were incubated at 37 °C and 5% CO_2_ for increasing lengths of time (0, 1, 2, 4, 8, and 24 hours), with subsequent Raman trapping analysis performed at each time-point. No changes in the EV Raman spectra or the deuterium peak intensities were observed over a period of 4 hours, indicating that both the EV composition and metabolic labelling remained stable over this period of time (ESI,† Fig. S6). While a small, but statistically significant, reduction in deuterium peak was observed at 8 and 24 hours, this peak remained readily observable, enabling Raman imaging of deuterium-labelled EVs at up to 24 hours post incubation. After confirming the stability of both EV composition and metabolic labelling, we sought to simultaneously image and characterise the EVs *in vitro via* confocal Raman spectroscopy. First, we incubated unlabelled MDA-MB-231 cells with unlabelled EVs for 4 hours to determine whether the addition of EVs had any effect on the Raman spectroscopic imaging and analysis. No deuterium signal was observed between 2025 and 2275 cm^−1^, as expected, while univariate identification of the subcellular components was maintained (ESI,† Fig. S7).

**Fig. 5:**
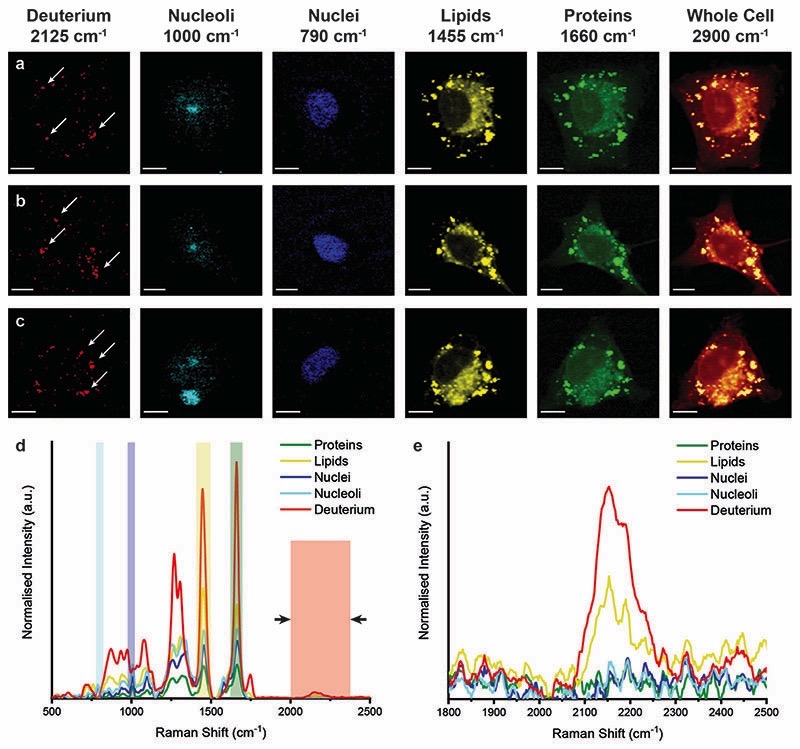
Raman spectroscopic imaging and univariate analysis of MDA-MB-231 cells after 4 hour incubation with EVs isolated from D_2_O-treated MDA-MB-231 cells. (a–c) Univariate images of normalised Raman spectroscopic image data for MDA-MB-231 cells (*n* = 3) incubated for 4 hours with EVs isolated from D_2_O MDA-MB-231 cells highlighting the distribution of different subcellular components including deuterium (red, 2025– 2275 cm^−^^1^), nucleoli (cyan, 985–1015 cm^−^^1^), nuclei (blue, 775–805 cm^−^^1^), lipids (yellow, 1425–1485 cm^−^^1^), proteins (green, 1635–1685 cm^−^^1^), and the whole cell (2800–3000 cm^−^^1^). Scale bars = 10 mm. White arrows indicate deuterium positive regions. (d) Exemplar Raman spectra and (e) magnified silent region of cell areas positive for deuterium, nucleoli, nuclei, lipids, and proteins (arrows indicate FWHM peak measurement, see Materials and methods for details).

However, when unlabelled cells were incubated with D_2_O labelled EVs, Raman spectroscopic imaging and subsequent univariate analysis indicated the presence of deuterium signal at discrete locations throughout the cytoplasm (Fig. 5). The observation of deuterium signal at discrete locations suggests that the EVs have not, at this point, been degraded, as upon degradation we would expect to see weak deuterium signal dispersed throughout the cell. The spectra at these locations contained the same protein and lipid signal hallmarks as observed for the D_2_O EVs in solution, in addition to signal contributed from the surrounding cell (ESI,† Fig. S5). As the size of the isolated EVs, measured at approximately 120 nm *via* NTA, is less than the applied Raman spectroscopic imaging resolution of 500 nm, the detected deuterium signal likely corresponds to clusters of EVs, possibly packaged within endosomes or lysosomes as indicated by prior imaging studies.^14,18^

**Fig. 6:**
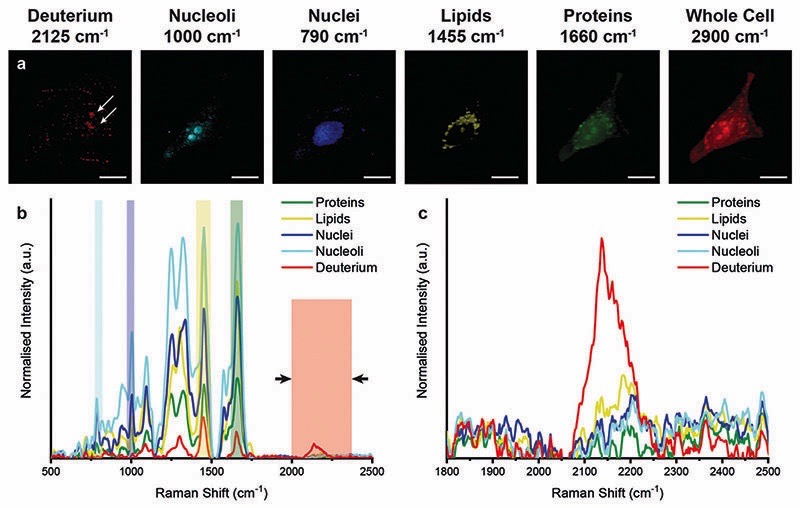
3D Raman spectroscopic imaging and univariate analysis of MDA-MB-231 cell after 4 hour incubation with EVs isolated from D_2_O-treated MDA-MB-231 cells. (a) Merged univariate image stacks of normalised Raman spectroscopic image data for MDA-MB-231 cell incubated for 4 hours with EVs isolated from D_2_O MDA-MB-231 cells highlighting the distribution of different subcellular components including deuterium (red, 2025–2275 cm^−^^1^), nucleoli (cyan, 985–1015 cm^−^^1^), nuclei (blue, 775–805 cm^−^^1^), lipids (yellow, 1425–1485 cm^−^^1^), and proteins (green, 1635–1685 cm^−^^1^). Scale bars = 10 mm. White arrows indicate deuterium positive regions. (b) Exemplar Raman spectra and (c) magnified silent region of cell areas positive for deuterium, nucleoli, nuclei, lipids, and proteins (arrows indicate FWHM peak measurement, see Materials and methods for details).

We then aimed to observe the distribution of EVs *in vitro* in 3D using our previously developed qVRI method for volumetric Raman imaging.^56^ Employing the confocality of our Raman microspectroscopy system, we acquired a 3D image of an MDA-MB-231 cell that had been incubated with D_2_O EVs for 4 hours (Fig. 6 and ESI,† Video S1). As in the 2D case, univariate analysis enabled spectroscopic identification of multiple sub-cellular components as well as the metabolically labelled EVs. These results thus demonstrate the unprecedented capacity of Raman spectroscopy to perform simultaneous imaging and characterisation of EVs *in vitro* in 2D and 3D.

Lastly, to highlight the potential of Raman spectroscopic molecular imaging of EVs, we demonstrated how the information rich Raman spectral images can be used to perform relative quantification and comparison of EV uptake. To do this, we incubated D_2_O EVs isolated from MDA-MB-231 cells for 4 hours with both non-malignant MCF-10A cells at 37 °C and with MDA-MB-231 at 4 °C. In each case, we hypothesised that we would observe a reduced uptake of D_2_O EVs relative to the MDA-MB-231 cells at 37 °C, due to the reported self-selectivity of the interactions governing EV uptake by cells and the inhibitory effects of incubation at 4 °C on EV uptake.^57–60^ In each case, we observed a decrease in deuterium signal inside the cells, though with increased deuterium signal at the cell surface, relative to the MDA-MB-231 cells (37 °C) (ESI,† Fig. S8 and S9).

**Fig. 7:**
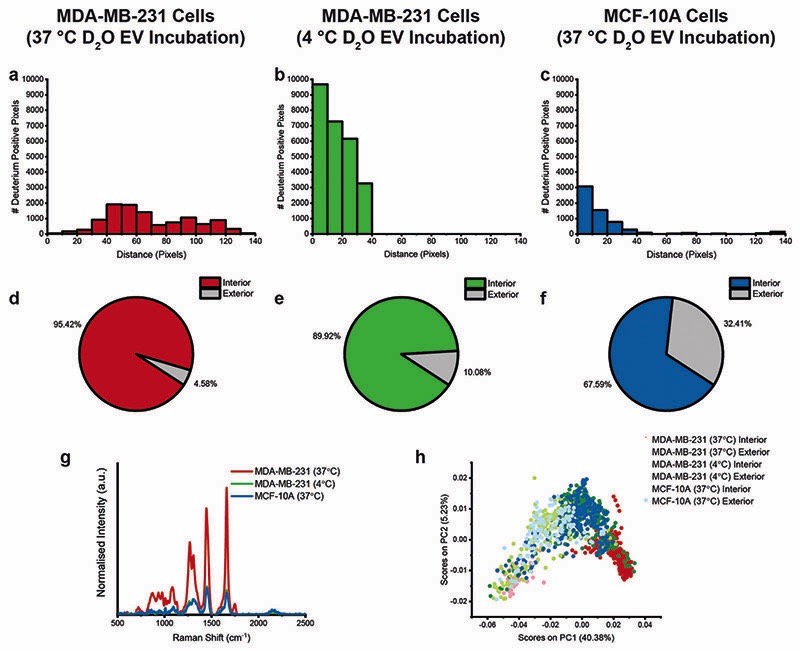
Raman spectroscopic analysis of D_2_O EV uptake for MDA-MB-231 cells and MCF-10A cells following 4 hour incubation at 37 °C or 4 °C with EVs isolated from D_2_O-treated MDA-MB-231 cells. (a–c) Distances of deuterium-positive pixels inside each cell from nearest cell membrane location for (a) MDA-MB-231 cells (37 °C), (b) MDA-MB-231 cells (4 °C), and (c) MCF-10A cells (37 °C) (*n* = 3 cells in each case). (d–f) Percentage of deuterium signal detected inside (or associated with) cell and outside of cell (*n* = 3 cells in each case). (g) Mean Raman spectra of deuterium-positive pixels inside (or associated with) each cell, by cell type, and (h) PCA scores for Raman spectra of deuterium-positive pixels for all cells combined (*n* = 3 cells in each case).

We next developed an automated Raman spectroscopic image processing and analysis framework to perform relative quantification and comparison of D_2_O EV uptake in cells across the different conditions. This framework, which takes corresponding univariate Raman images of the whole cell and deuterium as well as the Raman spectral information as inputs, automatically identifies the location of any deuterium signal within the cell to quantify EV uptake and internalisation distances (ESI,† Fig. S10). Using this framework, we identified that D_2_O EVs were located further into the interior of MDA-MB-231 cells (37 °C) as compared to the MDA-MB-231 cells (4 °C) and the MCF-10A cells (37 °C) (Fig. 7a–c). We also confirmed the increased proportion of deuterium signal inside (or associated with) the MDA-MB-231 cells (37 °C) relative to the other cell cohorts (Fig. 7d–f). Analysis of the Raman spectra for the deuterium-positive pixels identified in each case provided further insights into the EV uptake across cohorts. The Raman spectra for the internalised deuterium pixels of the MDA-MB-231 cells (37 °C) displayed increased protein and lipid peaks relative to the other cell cohorts, likely due to the background signature of cellular components in the Raman confocal volume (Fig. 7g). In contrast, the deuterium signal for the MDA-MB-231 cells (4 °C) and MCF-10A cells (37 °C) displayed an increased proportion of deuterium signal and a corresponding reduction in protein and lipid signatures, suggesting that the deuterium signal in these cases arises from EVs bound to the cell surface rather than internalised by the cells. PCA separation of the deuterium Raman spectral signatures clearly distinguishes between the internal deuterium spectra for the MDA-MB-231 cells (37 °C) and the deuterium spectra for the MDA-MB-231 cells (4 °C) and MCF-10A cells (37 °C) (Fig. 7h and ESI,† Fig. S11, S12). Together, these results highlight the potential for the exploitation of the high information content Raman spectral images to gain a deeper understanding of the biological mechanisms underpinning EVs.

EVs have demonstrated exciting potential as biomarkers for numerous pathological conditions as well as for bioengineerable drug delivery vectors, sparking much research across these areas.^11,61^ However, despite the considerable interest in EVs, much of the details surrounding their biogenesis, secretion, targeting, uptake, and cargo release remains poorly understood.^1^ The inherent heterogeneity of EVs hampers understanding of these biological mechanisms. Further, as the biological compositions of EVs reflect their parent cells, it is difficult to observe EVs both *in vitro* and *in vivo* without the use of labelling strategies that potentially alter their native functionality. As the complex mechanisms underlying both the targeting and uptake of EVs appear to depend largely on the EV composition, it is essential to employ strategies for both imaging and analysis that do not significantly disrupt this makeup. This is particularly relevant given that the effects of compositional changes due to existing labelling strategies such as lipophilic dye labelling or surface functionalisation remain unclear.^1,32^

The Raman spectroscopic metabolic labelling methodology we present here represents an exciting advance in the *in vitro* imaging and characterisation of EVs. Our approach permits the acquisition of information-rich whole-cell spectroscopic data, for both direct EV visualisation and compositional analysis in 2D and 3D through a minimally obstructive metabolic labelling strategy. Importantly, this EV-specific metabolic labelling strategy enables compositional analysis *in vitro*, which is not otherwise possible due to the cellular origins of EVs, which gives them a similar biochemical makeup to the surrounding cellular environment. This analysis reveals the lipid rich makeup of EVs as well as the presence of protein peaks, agreeing with the Raman trapping analysis of isolated EVs in solution and previous studies that indicate that EVs consist of a range of lipids, proteins, and nucleic acid cargoes.^1,61^ As the biochemical makeup of EVs is now known to depend on both the cellular origin and its physiological state, the molecular characterisation enabled by this technique could provide important insights into EV biology and thus help to guide the design of future EV therapeutic systems.^62^ The automated Raman spectroscopic image processing and analysis framework presented can further propel comparative studies of EV uptake under different conditions or for different cell types. This would potentially provide further insights into the roles of different molecules for EV cellular targeting and uptake, with further relevance for the design of EV therapeutics.

While the potential for simultaneous imaging and molecular characterisation of EVs *in vitro* using this technique is unparalleled by existing EV imaging strategies, there are some limitations that need to be overcome before it can be more widely employed. Firstly, while Raman spectroscopy provides a wealth of biochemical information, unpacking this information to identify particular lipid, protein, or nucleic acid components within EVs will require more sophisticated analysis. Multi-variate analyses and spectral unmixing techniques are likely to provide more information when examining EVs from multiple different sources, and correlation with other techniques such as mass spectrometry would likely allow more advanced lipidomic characterisation of EVs.^24^ Secondly, high resolution Raman spectroscopic imaging is currently too slow to permit high-throughput cell studies, thus limiting our present study to fixed cell imaging. However, recent advances in hyper-spectral stimulated Raman spectroscopic imaging open the possibility for real time tracking of EVs, thus providing further insights into EV trafficking and enable high throughput *in vitro* EV studies.^63,64^ Used together, these strategies would provide comprehensive real time molecular characterisation and imaging of EVs *in vitro* to advance our understanding of EV biology.

## Conclusions

The combination of Raman spectroscopic imaging with the molecular labelling of key biomolecules with Raman-active reporter molecules opens up exciting opportunities to study EV biogenesis, uptake, and therapeutic delivery under a variety of normal and dysregulated cell states. The selective metabolic labelling of nucleic acids,^34^ amino acids,^39^ and key EV proteins such as the tetraspanins^65^ could enable further understanding of EV compositional changes that occur in response to different stimuli, facilitating advances in EV production and clinical application as therapeutic drug delivery vehicles.

## Supplementary Material

^†^Electronic supplementary information (ESI) available: https://github.com/conor-horgan/EV-Raman-Analysis

Supplementary Information

Supplementary movie

## References

[1] Niel GV, D’Angelo G, Raposo G (2018). Shedding light on the cell biology of extracellular vesicles. Nat Rev Mol Cell Biol.

[2] Simons M, Raposo G (2009). Exosomes – vesicular carriers for intercellular communication. Curr Opin Cell Biol.

[3] Armstrong JPK, Holme MN, Stevens MM (2017). Re-engineering extracellular vesicles as smart nanoscale therapeutics. ACS Nano.

[4] Cui S, Cheng Z, Qin W, Jiang L (2018). Exosomes as a liquid biopsy for lung cancer. Lung Cancer.

[5] Garcia-Romero N (2018). Extracellular vesicles compartment in liquid biopsies: clinical application. Mol Aspects Med.

[6] De Jong OG, Van Balkom BWM, Schiffelers RM, Bouten CVC, Verharr MC (2014). Extracellular vesicles: potential roles in regenerative medicine. Front Immunol.

[7] Luan X (2017). Engineering exosomes as refined biological nanoplatforms for drug delivery. Acta Pharmacol Sin.

[8] Kibria G, Ramos EK, Wan Y, Gius DR, Liu H (2018). Exosomes as a drug delivery system in cancer therapy: potential and challenges. Mol Pharmacol.

[9] Fuhrmann G, Herrmann IK, Stevens MM (2015). Cell-derived vesicles for drug therapy and diagnostics: opportunities and challenges. Nano Today.

[10] Théery C, Zitvogel L, Amigorena S (2002). Exosomes: composition, biogenesis and function. Rev Immunol.

[11] Armstrong JPK, Stevens MM (2018). Strategic design of extracellular vesicle drug delivery systems. Adv Drug Delivery Rev.

[12] Kim SY, Khanal D, Kalionis B, Chrzanowski W (2019). High-fidelity probing of the structure and heterogeneity of extracellular vesicles by resonance-enhanced atomic force microscopy infrared spectroscopy. Nat Protoc.

[13] Ohno SI (2013). Systemically injected exosomes targeted to EGFR deliver antitumor microRNA to breast cancer cells. Mol Ther.

[14] Chen C (2016). Imaging and intracellular tracking of cancer-derived exosomes using single-molecule localization-based super-resolution microscope. ACS Appl Mater Interfaces.

[15] Smyth TJ, Redzic JS, Graner MW, Anchordoquy TJ (2014). Examination of the specificity of tumor cell derived exosomes with tumor cells in vitro. Biochim Biophys Acta.

[16] Wang M, Altinoglu S, Takeda YS, Xu Q (2015). Integrating protein engineering and bioorthogonal click conjugation for extracellular vesicle modulation and intracellular delivery. PLoS One.

[17] Stickney Z, Losacco J, McDevitt S, Zhang Z, Lu B (2016). Development of exosome surface display technology in living human cells. Biochem Biophys Res Commun.

[18] Heusermann W (2016). Exosomes surf on filopodia to enter cells at endocytic hot spots, traffic within endosomes, and are targeted to the ER. J Cell Biol.

[19] Morales-Kastresana A (2017). Labeling extracellular vesicles for nanoscale flow cytometry. Sci Rep.

[20] Lassailly F, Griessinger E, Bonnet D (2010). Microenvironmental contaminations” induced by fluorescent lipophilic dyes used for non-invasive. Blood.

[21] Tario D, Muirhead KA, Pan D, Munson ME, Wallace K (2007). Tracking immune cell proliferation and cytotoxic potential using flow cytometry. Methods Mol Biol.

[22] Lai CP (2015). Visualization and tracking of tumour extracellular vesicle delivery and RNA translation using multiplexed reporters. Nat Commun.

[23] Kowal J (2016). Proteomic comparison defines novel markers to characterize heterogeneous populations of extracellular vesicle subtypes. Proc Natl Acad Sci U S A.

[24] Bergholt MS (2018). Correlated heterospectral lipidomics for biomolecular profiling of remyelination in multiple sclerosis. ACS Cent Sci.

[25] Butler J (2016). Using Raman spectroscopy to characterise biological materials. Nat Protoc.

[1] Evans CL (2005). Chemical imaging of tissue in vivo with video-rate coherent anti-Stokes Raman scattering microscopy. Proc Natl Acad Sci U S A.

[27] Gentleman E (2009). Comparative materials differences revealed in engineered bone as a function of cell-specific differentiation. Nat Mater.

[28] Lu F-K (2015). Label-free DNA imaging in vivo with stimulated Raman scattering microscopy. Proc Natl Acad Sci U S A.

[29] Penders J (2018). Single Particle Automated Raman Trapping Analysis. Nat Commun.

[30] Puppels GJ (1990). Studying single living cells and chromosomes by confocal Raman microspectroscopy. Nature.

[31] Klein K (2012). Label-free live-cell imaging with confocal Raman microscopy. Biophys J.

[32] Zhao Z, Shen Y, Hu F, Min W (2017). Applications of vibrational tags in biological imaging by Raman microscopy. Analyst.

[33] Wei L (2016). Live-cell bioorthogonal chemical imaging: stimulated Raman scattering microscopy of vibrational probes. Acc Chem Res.

[34] Wei L (2014). Live-cell imaging of alkyne-tagged small biomolecules by stimulated Raman scattering. Nat Methods.

[1] Long R (2017). Two-color vibrational imaging of glucose metabolism using stimulated Raman scattering. Chem Commun.

[36] Li J, Cheng JX (2014). Direct visualization of de novo lipogenesis in single living cells. Sci Rep.

[37] Berry D (2015). Tracking heavy water (D_2_O) incorporation for identifying and sorting active microbial cells. Proc Natl Acad Sci U S A.

[38] Chernenko T (2012). Raman microscopy for noninvasive imaging of pharmaceutical nanocarriers: intracellular distribution of cationic liposomes of different composition. Mol Pharmacol.

[39] Hu F, Lamprecht MR, Wei L, Morrison B, Min W (2016). Bioorthogonal chemical imaging of metabolic activities in live mammalian hippocampal tissues with stimulated Raman scattering. Sci Rep.

[40] Tipping WJ, Lee M, Serrels A, Brunton VG, Hulme AN (2016). Stimulated Raman scattering microscopy: an emerging tool for drug discovery. Chem Soc Rev.

[41] Yamakoshi H (2012). Alkyne-tag Raman imaging for visualization of mobile small molecules in live cells alkyne-tag Raman imaging for visualization of mobile small molecules in live cells. J Am Chem Soc.

[42] Matthäus C, Kale A, Chernenko T, Torchilin V, Diem M (2008). New ways of imaging uptake and intracellular fate of liposomal drug carrier systems inside individual cells, based on Raman microscopy. Mol Pharmacol.

[43] Huser T, Chan J (2015). Raman spectroscopy for physiological investigations of tissues and cells. Adv Drug Delivery Rev.

[44] Gualerzi A (2017). Raman spectroscopy uncovers biochemical tissue-related features of extracellular vesicles from mesenchymal stromal cells. Sci Rep.

[45] Smith ZJ (2015). Single exosome study reveals subpopulations distributed among cell lines with variability related to membrane content. J Extracell Vesicles.

[46] Tatischeff I, Larquet E, Falcón-Pérez JM, Turpin P-Y, Kruglik SG (2012). Fast characterisation of cell-derived extra-cellular vesicles by nanoparticles tracking analysis, cryo-electron microscopy, and Raman tweezers microspectroscopy. Extracell Vesicles.

[47] Gualerzi A (2019). Raman spectroscopy as a quick tool to assess purity of extracellular vesicle preparations and predict their functionality. J Extracell Vesicles.

[48] Théry C (2018). Minimal information for studies of extra-cellular vesicles 2018 (MISEV2018): a position statement of the International Society for Extracellular Vesicles and update of the MISEV2014 guidelines. J. Extracell. Vesicles.

[49] Kushner DJ, Baker A, Dunstall TG (1999). Pharmacological uses and perspectives of heavy water and deuterated compounds. Can J Physiol Pharmacol.

[50] Draux F (2009). Raman spectral imaging of single living cancer cells: a preliminary study. Analyst.

[51] Movasaghi Z, Rehman S, Rehman IU (2007). Raman Spectroscopy of Biological Tissues. Appl Spectrosc Rev.

[52] Schulze G, Konorov SO, Piret JM, Blades MW, Turner RFB (2013). Label-free imaging of mammalian cell nucleoli by Raman microspectroscopy. Analyst.

[53] Czamara K (2015). Raman spectroscopy of lipids: a review. Raman Spectrosc.

[54] Stone N (2004). Raman spectroscopy for identification of epithelial cancers. Faraday Discuss.

[55] Hu F, Wei L, Zheng C, Shen Y, Min W (2014). Live-cell vibrational imaging of choline metabolites by stimulated Raman scattering coupled with isotope-based metabolic labeling. Analyst.

[56] Kallepitis C (2017). Quantitative volumetric Raman imaging of three dimensional cell cultures. Nat Commun.

[57] Raposo G, Stoorvogel W (2013). Extracellular vesicles: Exosomes, microvesicles, and friends. J Cell Biol.

[58] Rana S, Yue S, Stadel D, Zöller M (2012). Toward tailored exosomes: the exosomal tetraspanin web contributes to target cell selection. J Biochem Cell Biol.

[59] Escrevente C, Keller S, Altevogt P, Costa J (2011). Interaction and uptake of exosomes by ovarian cancer cells. BMC Cancer.

[60] Tian T, Wang Y, Wang H, Zhu Z, Xiao Z (2010). Visualizing of the cellular uptake and intracellular trafficking of exosomes by live-cell microscopy. J Cell Biochem.

[61] Toro JD, Herschlik L, Waldner C, Mongini C (2015). Emerging roles of exosomes in normal and pathological conditions: new insights for diagnosis and. Front Immunol.

[62] Colombo M, Raposo G, Théry C (2014). Biogenesis, Secretion, and Intercellular Interactions of Exosomes and Other Extracellular Vesicles. Annu Rev Cell Dev Biol.

[63] Fu D, Holtom G, Freudiger C, Zhang X, Xie XS (2013). Hyperspectral imaging with stimulated Raman scattering by chirped femtosecond lasers. J Phys Chem B.

[64] Fu D (2014). Imaging the intracellular distribution of tyrosine kinase inhibitors in living cells with quantitative hyperspectral stimulated Raman scattering. Nat Chem.

[65] Andreu Z, Yáñez-Mó M (2014). Tetraspanins in extracellular vesicle formation and function. Immunol.

