# Supplementary material for: Molecular imaging of extracellular vesicles in vitro via Raman metabolic labelling†
Source: J Mater Chem B. Author manuscript; Available in PMC 2021 May 17. (PMC7610785; doi:10.1039/d0tb00620c)
Supplement: Supplementary Information [file EMS103713-supplement-Supplementary_Information.pdf]

Electronic Supplementary Material (ESI) for Journal of Materials Chemistry B.
This journal is © The Royal Society of Chemistry 2020

Molecular Imaging of Extracellular Vesicles *In Vitro* via Raman Metabolic Labelling

Conor C. Horgan,[a,b,c†] Anika Nagelkerke,[a,b,c†] Thomas E. Whittaker,[a,b,c] Valeria Nele,[a,b,c] Lucia Massi,[a,b,c] Ulrike Kauscher,[a,b,c] Jelle Penders,[a,b,c] Mads S. Bergholt,[a,b,c] Steve R. Hood[d] and Molly M. Stevens*[a,b,c]

Supplementary Information

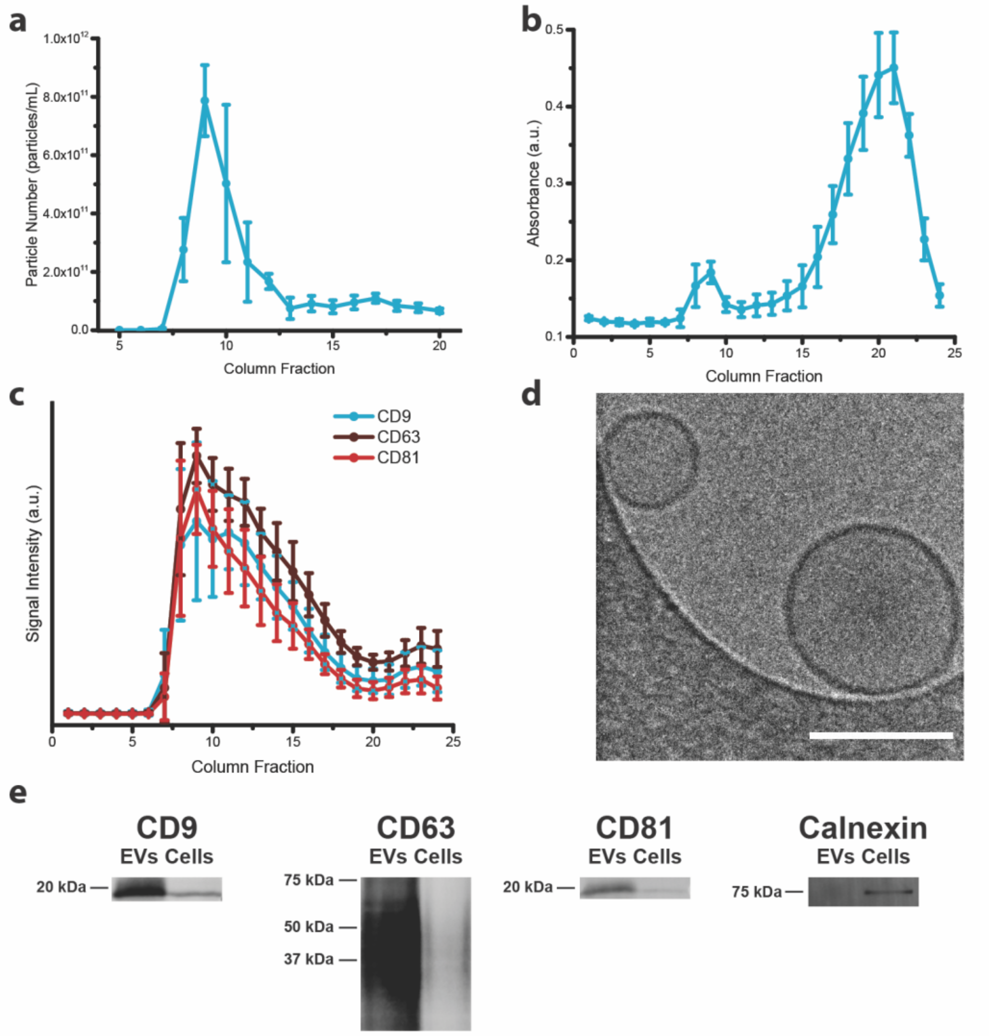

Supplementary Figure 1 | Characterization of EV isolation using ultrafiltration and size exclusion chromatography from conditioned medium from MDA-MB-231 control cells, serum starved for 72 hours. (**a**) NTA on individual fractions from column trace obtained from size exclusion chromatography. (N = 3, n = 3). Data presented as mean ± SD. (**b**) Analysis of protein quantity from column trace obtained from SEC. (N = 3, n = 3). Data presented as mean ± SD. (**c**) Analysis of fractions obtained from SEC for expression of the three EV markers CD9, CD63 and CD81. (N = 6, n = 1). Data presented as mean ± SD. (**d**) Cryo-TEM image of purified vesicles. Scale bar = 100 nm. (**e**) Western blot analysis of cells and purified EVs for expression of EV markers CD9, CD63 and CD81, as well as the endoplasmic reticulum protein Calnexin.

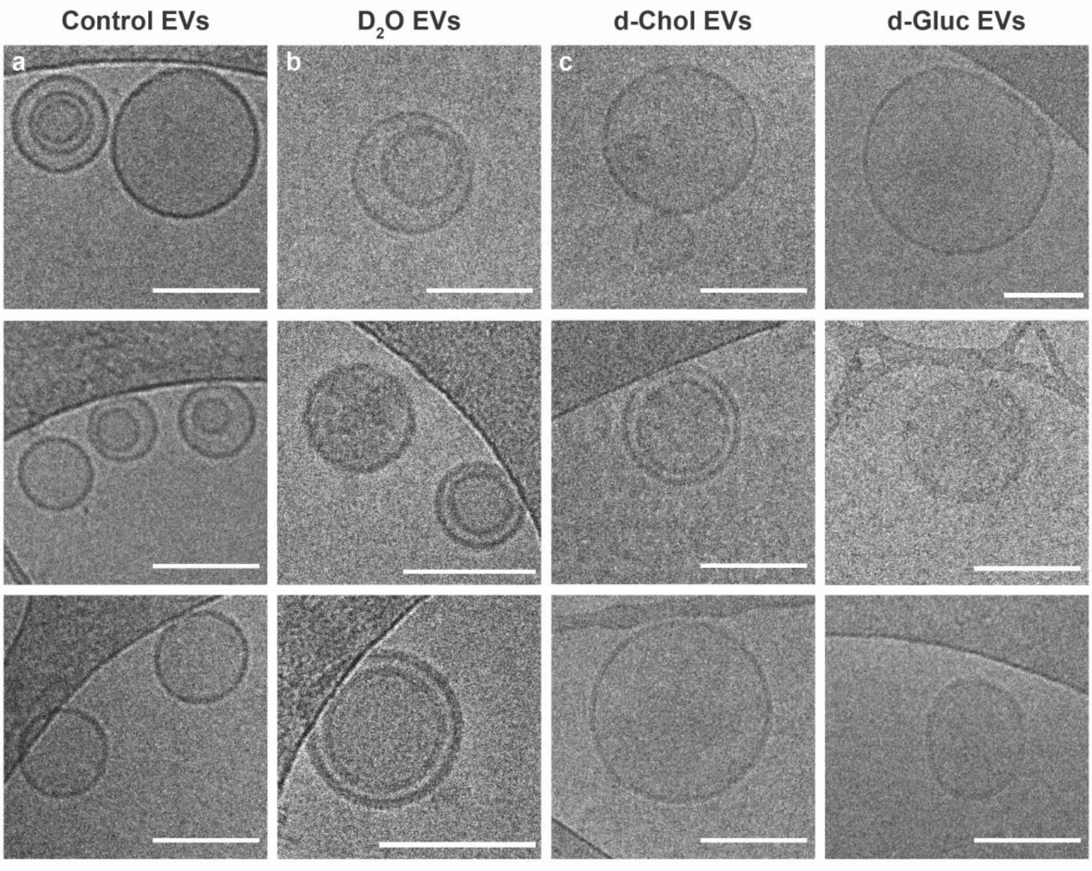

Supplementary Figure 2 | Cryo-TEM images of EVs metabolically labelled with deuterium. (**a-d**) Cryo-TEM images of EVs isolated from (**a**) control MDA-MB-231 cells, (**b**) D_2O MDA-MB-231 cells, (**c**) d-Chol MDA-MB-231 cells, or (**d**) d-Gluc MDA-MB-231 cells. Scale bars = 100 nm.

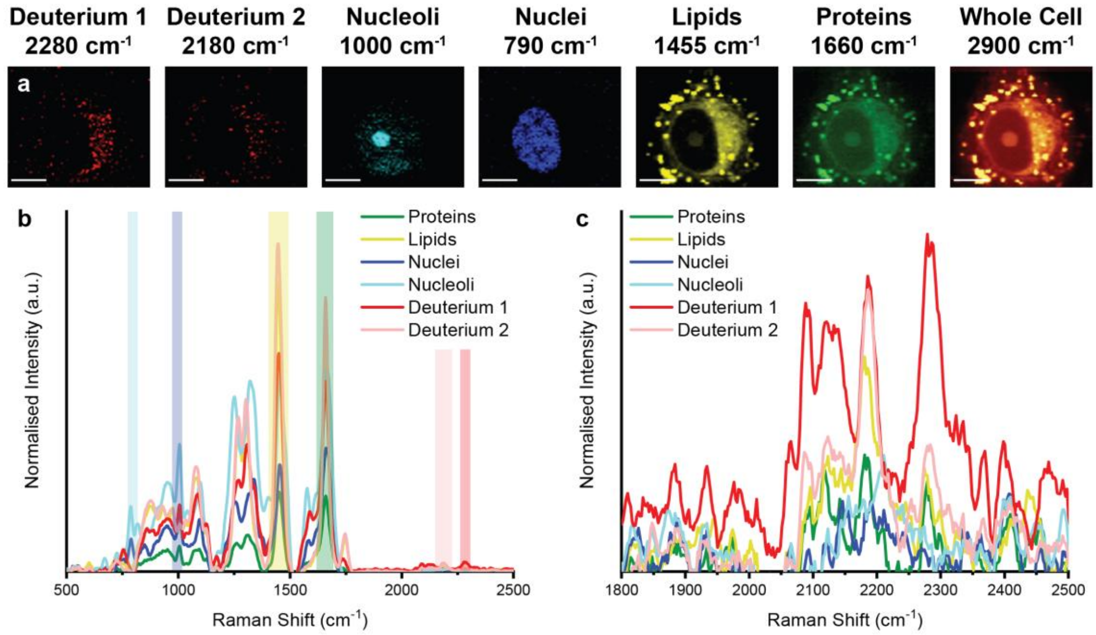

Supplementary Figure 3 | Raman spectroscopic imaging and univariate analysis of d-Chol-treated MDA-MB-231 cells. (**a**) Univariate images of normalised Raman spectroscopic image data for an MDA-MB-231 cell after incubation in d-Chol-containing medium for 72 hours highlighting the distribution of different subcellular components including deuterium peak 1 (red, 2262.5 – 2297.5 cm^{-1}), deuterium peak 2 (red, 2155 – 2205 cm^{-1}), nucleoli (cyan, 985 – 1015 cm^{-1}), nuclei (blue, 775 – 805 cm^{-1}), lipids (yellow, 1425 – 1485 cm^{-1}), proteins (green, 1635 – 1685 cm^{-1}), and the whole cell (2800 – 3000 cm^{-1}). Scale bars = 10 µm. (**b**) Exemplar Raman spectra and (**c**) magnified silent region of cell areas positive for deuterium, nucleoli, nuclei, lipids, and proteins.

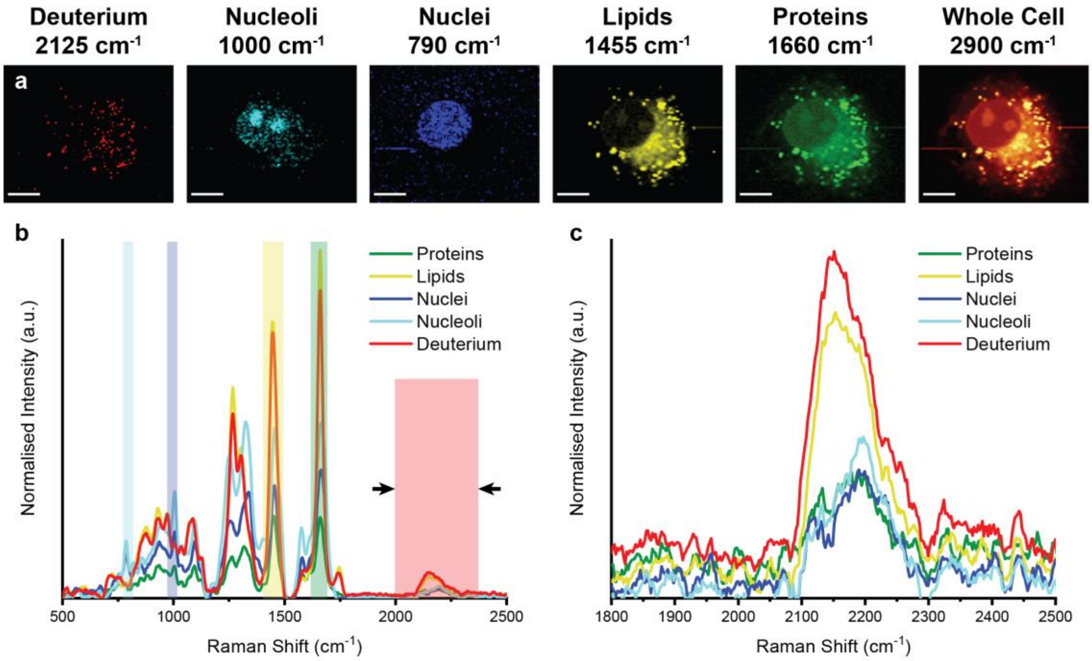

Supplementary Figure 4 | Raman spectroscopic imaging and univariate analysis of d-Gluc-treated MDA-MB-231 cells. (**a**) Univariate images of normalised Raman spectroscopic image data for an MDA-MB-231 cell after incubation in d-Gluc-containing medium for 72 hours highlighting the distribution of different subcellular components including deuterium (red, 2025 – 2275 cm^{-1}), nucleoli (cyan, 985 – 1015 cm^{-1}), nuclei (blue, 775 – 805 cm^{-1}), lipids (yellow, 1425 – 1485 cm^{-1}), proteins (green, 1635 – 1685 cm^{-1}), and the whole cell (2800 – 3000 cm^{-1}). Scale bars = 10 µm. (**b**) Exemplar Raman spectra and (**c**) magnified silent region of cell areas positive for deuterium, nucleoli, nuclei, lipids, and proteins (arrows indicate FWHM peak measurement, see Materials and Methods for details).

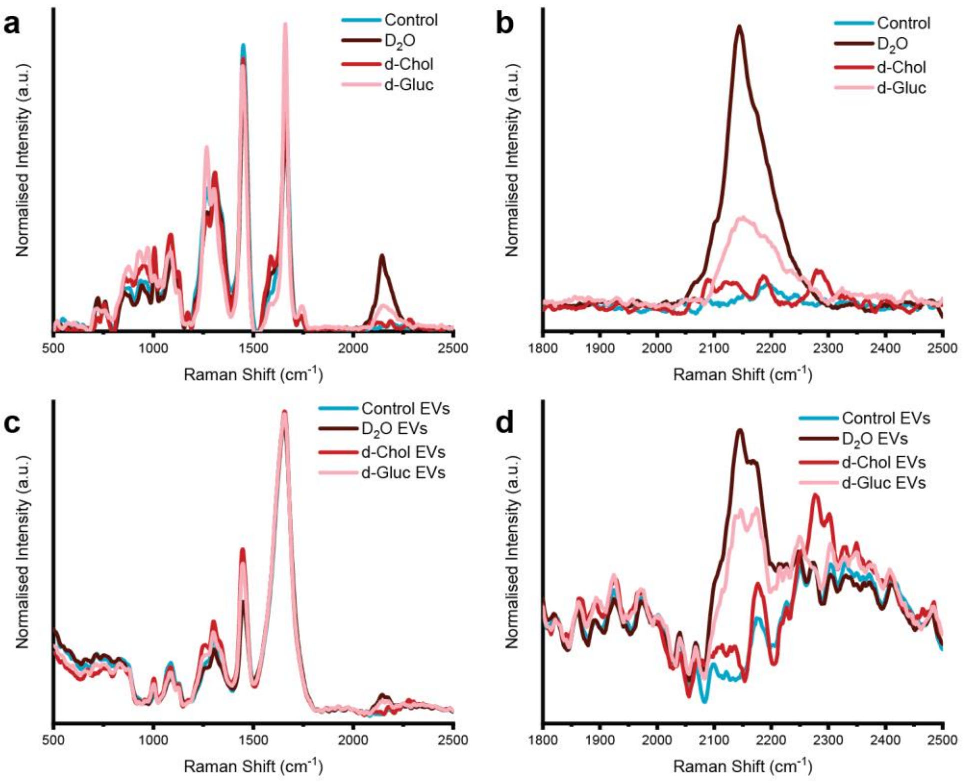

Supplementary Figure 5 | Raman spectral analysis of deuterium metabolic incorporation in cells and in isolated EVs *in vitro*. (**a**) Exemplar Raman spectra and (**b**) magnified silent region for control, D_2O, d-Chol, and d-Gluc MDA-MB-231 cells. (**c**) Mean Raman spectra and (**d**) magnified silent region for optically trapped EVs isolated from control, D_2O, d-Chol, and d-Gluc MDA-MB-231 cells (N = 3, n = 15).

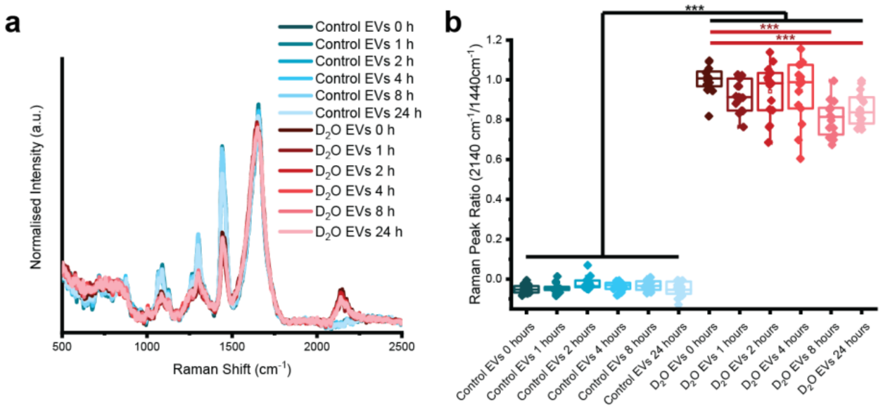

Supplementary Figure 6 | Stability of EV metabolic labelling with deuterium. (**a**) Normalised Raman spectra of control and D_2O EVs incubated at 37 ˚C and 5% CO_2 after isolation for increasing lengths of time (n = 15). (**b**) Ratio of the Raman spectral peak at 2140 cm^{-1} (C-D) to the peak at 1440 cm^{-1} (C-H) (n = 15) (One-way ANOVA, Tukey honest significant differences post hoc correction, * $P < 0.05$, ** $P < 0.01$, *** $P < 0.001$).

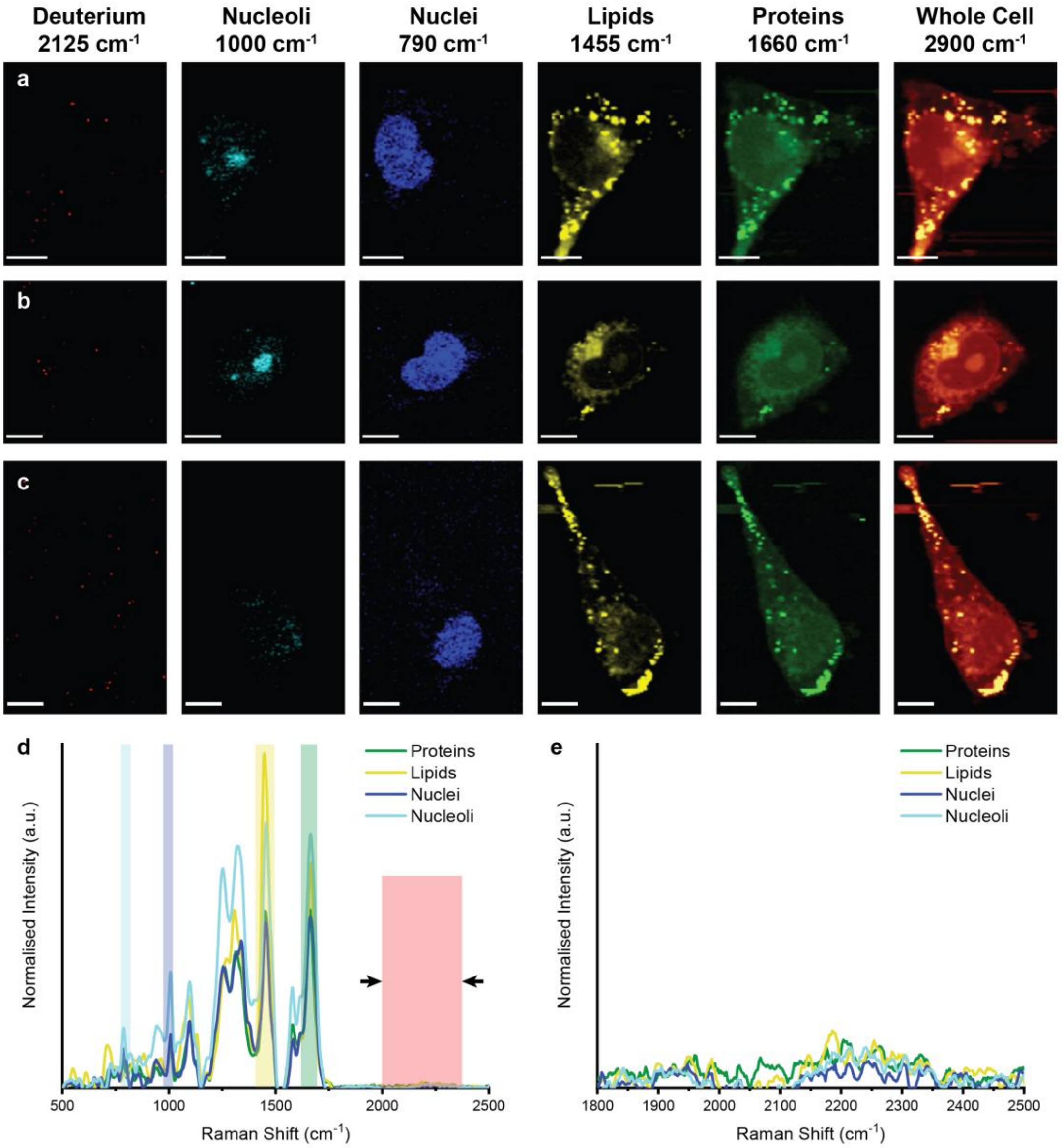

Supplementary Figure 7 | Raman spectroscopic imaging and univariate analysis of MDA-MB-231 cells after 4 hour incubation at 37 ˚C with EVs isolated from control MDA-MB-231 cells. (**a-c**) Univariate images of normalised Raman spectroscopic image data for MDA-MB-231 cells (n = 3) incubated for 4 hours at 37 ˚C with EVs isolated from control MDA-MB-231 cells highlighting the distribution of different subcellular components including deuterium (red, 2025 – 2275 cm^{-1}), nucleoli (cyan, 985 – 1015 cm^{-1}), nuclei (blue, 775 – 805 cm^{-1}), lipids (yellow, 1425 – 1485 cm^{-1}), proteins (green, 1635 – 1685 cm^{-1}), and the whole cell (2800 – 3000 cm^{-1}). Scale bars = 10 µm. (**d**) Exemplar Raman spectra and (**e**) magnified silent region of cell areas positive for nucleoli, nuclei, lipids, and proteins (arrows indicate FWHM peak measurement, see Materials and Methods for details).

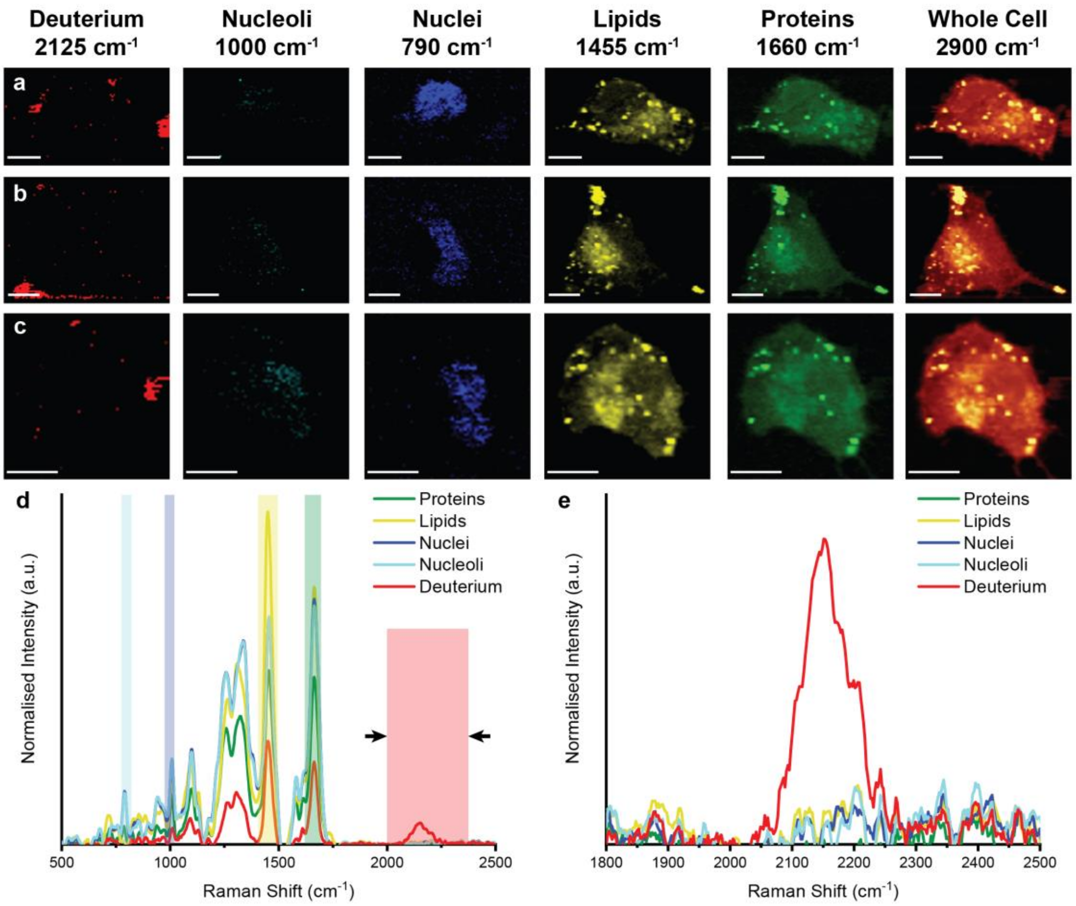

Supplementary Figure 8 | Raman spectroscopic imaging and univariate analysis of MDA-MB-231 cells after 4 hour incubation at 4 ˚C with EVs isolated from D_2O-treated MDA-MB-231 cells. (**a-c**) Univariate images of normalised Raman spectroscopic image data for MDA-MB-231 cells (n = 3) incubated for 4 hours at 4 ˚C with EVs isolated from D_2O MDA-MB-231 cells highlighting the distribution of different subcellular components including deuterium (red, 2025 cm^{-1} – 2275 cm^{-1}), nucleoli (cyan, 985 – 1015 cm^{-1}), nuclei (blue, 775 – 805 cm^{-1}), lipids (yellow, 1425 – 1485 cm^{-1}), proteins (green, 1635 – 1685 cm^{-1}), and the whole cell (2800 – 3000 cm^{-1}). Scale bars = 10 µm. White arrows indicate deuterium positive regions. (**d**) Exemplar Raman spectra and (**e**) magnified silent region of cell areas positive for deuterium, nucleoli, nuclei, lipids, and proteins (arrows indicate FWHM peak measurement, see Materials and Methods for details).

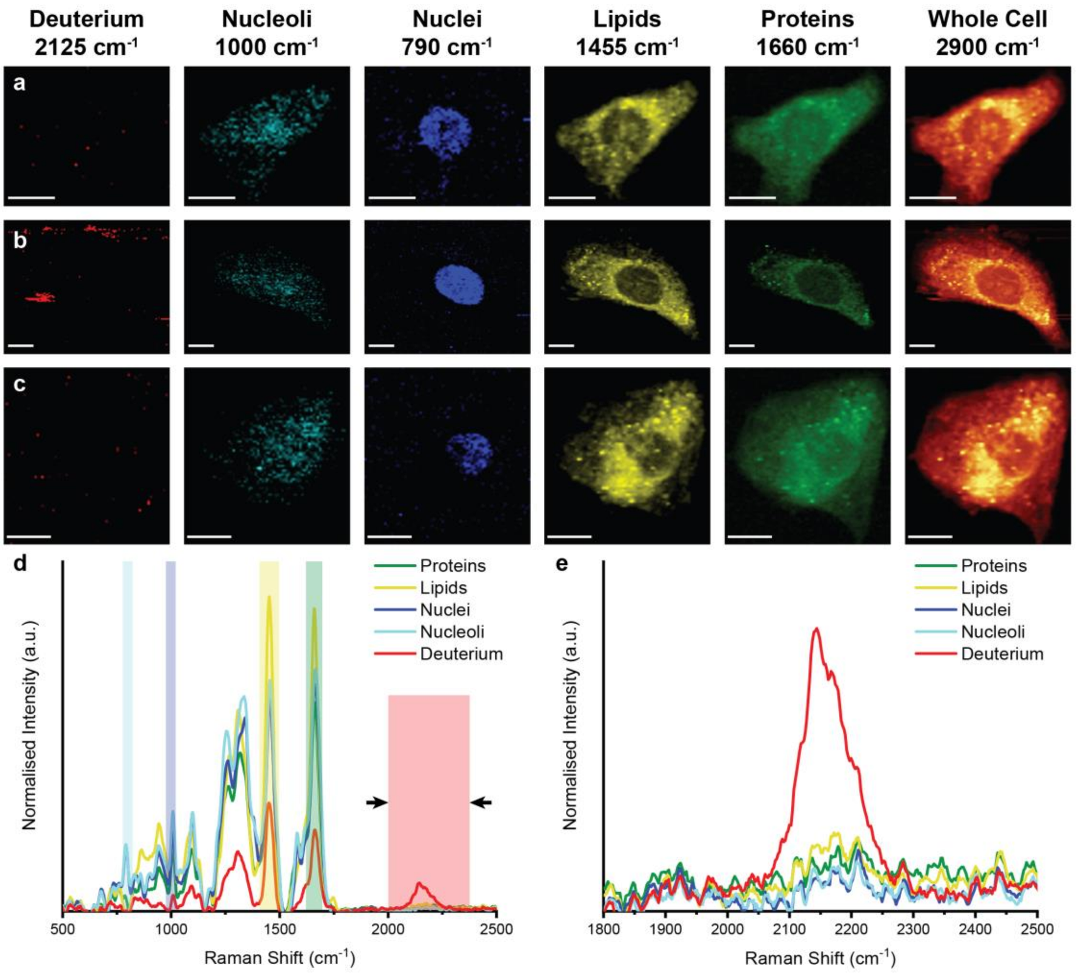

Supplementary Figure 9 | Raman spectroscopic imaging and univariate analysis of MCF-10A cells after 4 hour incubation at 37 ˚C with EVs isolated from D_2O-treated MCF-10A cells. (**a-c**) Univariate images of normalised Raman spectroscopic image data for MCF-10A cells (n = 3) incubated for 4 hours at 37 ˚C with EVs isolated from D_2O MDA-MB-231 cells highlighting the distribution of different subcellular components including deuterium (red, 2025 cm^{-1} – 2275 cm^{-1}), nucleoli (cyan, 985 – 1015 cm^{-1}), nuclei (blue, 775 – 805 cm^{-1}), lipids (yellow, 1425 – 1485 cm^{-1}), proteins (green, 1635 – 1685 cm^{-1}), and the whole cell (2800 – 3000 cm^{-1}). Scale bars = 10 µm. White arrows indicate deuterium positive regions. (**d**) Exemplar Raman spectra and (**e**) magnified silent region of cell areas positive for deuterium, nucleoli, nuclei, lipids, and proteins (arrows indicate FWHM peak measurement, see Materials and Methods for details).

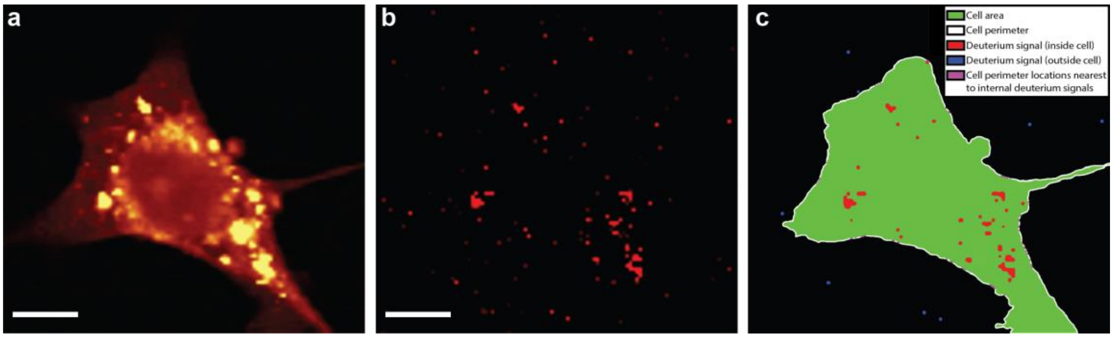

Supplementary Figure 10 | Raman spectral image analysis of D_2O EVs in cells. (**a**) Example whole cell image for a single MDA-MB-231 cell, (**b**) the corresponding univariate deuterium image, and (**c**) automated analysis result after passing (**a**), (**b**), and the corresponding Raman spectral information to the analysis script (see online supporting information), resulting in identification of the cell area (green), cell perimeter (white), deuterium signal inside the cell (red), deuterium signal outside the cell (blue), and the cell perimeter locations nearest to each of the internal deuterium signals (purple).

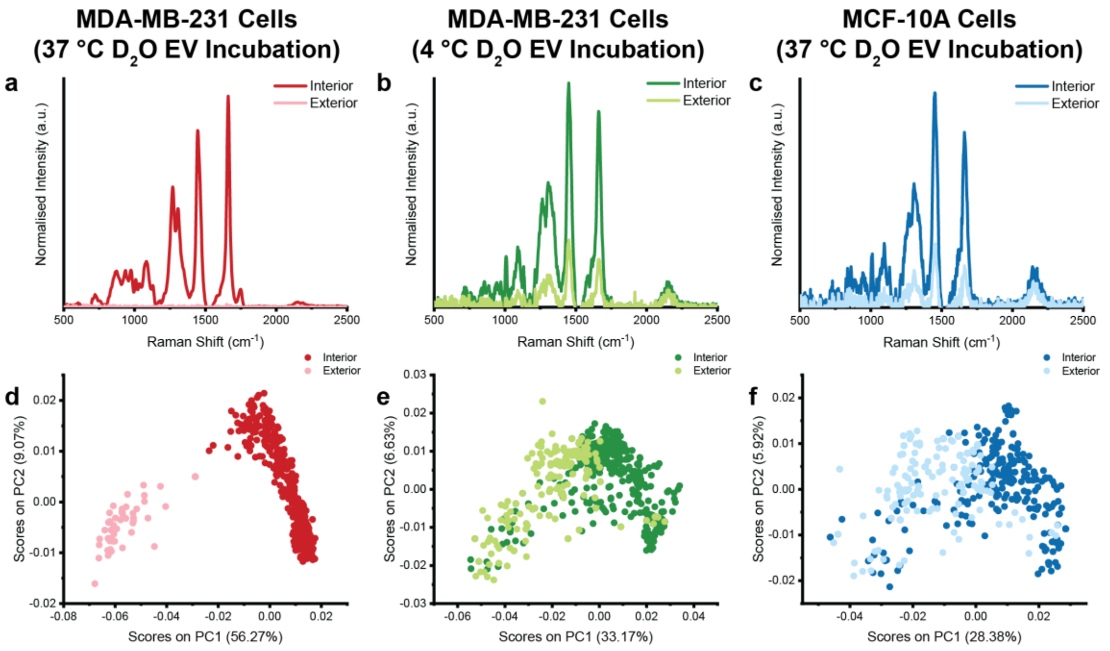

Supplementary Figure 11 | Raman spectral comparative analysis of deuterium-positive pixels for all cells. (**a-c**) Mean Raman spectra for deuterium signal detected inside (or associated with) cell and outside of cell (n = 3 cells in each case). (**d-f**) PCA scores for Raman spectra of deuterium signal detected inside (or associated with) cells and outside of cells (n = 3 cells in each case).

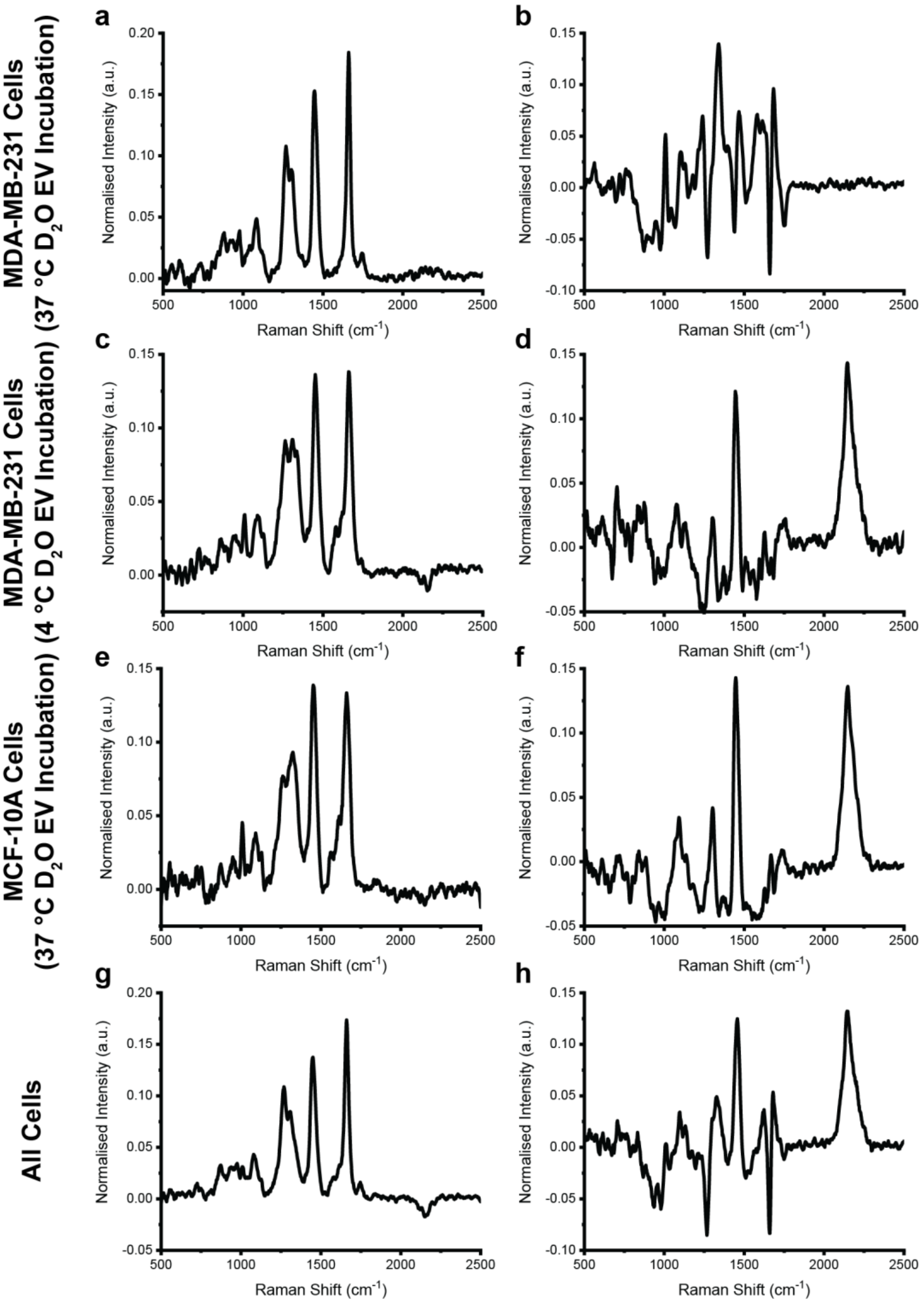

Supplementary Figure 12 | PCA principal components for Raman spectra of deuterium-positive pixels for MDA-MB-231 cells and MCF-10A cells following 4 hour incubation at 37 ˚C or 4 ˚C with EVs isolated from D_2O-treated MDA-MB-231 cells. PCA principal components for Raman spectra of deuterium-positive pixels for (**a, b**) MDA-MB-231 cells incubated with D_2O EVs at 37 ˚C, (**c, d**) MDA-MB-231 cells incubated with D_2O EVs at 4 ˚C, (**e, f**) MCF-10A cells incubated with D_2O EVs at 37 ˚C, and (**g, h**) all cells combined (n = 3 cells in each case).

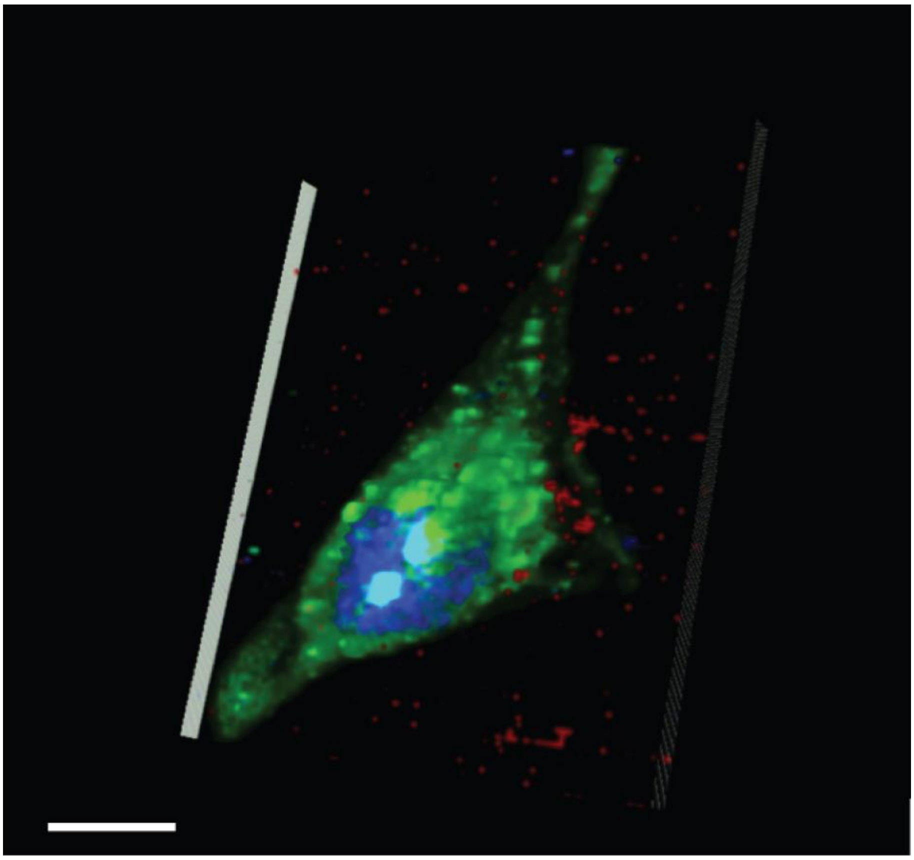

Supplementary Video 1 | 3D Raman spectroscopic imaging and univariate analysis of MDA-MB-231 cell after 4 hour incubation at 37 ˚C with EVs isolated from D_2O-treated MDA-MB-231 cells. 3D rotation of merged univariate image stacks of normalised Raman spectroscopic image data for MDA-MB-231 cell incubated for 4 hours at 37 ˚C with EVs isolated from D_2O MDA-MB-231 cells highlighting the distribution of different subcellular components including deuterium (red, 2025 cm^{-1} – 2275 cm^{-1}), nucleoli (cyan, 985 – 1015 cm^{-1}), nuclei (blue, 775 – 805 cm^{-1}), lipids (yellow, 1425 – 1485 cm^{-1}), and proteins (green, 1635 – 1685 cm^{-1}). Scale bar = 10 µm.
